# Supplementary material for: Chronic disaster impact: the long-term psychological and physical health consequences of housing damage due to induced earthquakes
Source: BMJ Open. 2021 May 5;11(5):e040710. doi: 10.1136/bmjopen-2020-040710 (PMC8103378; doi:10.1136/bmjopen-2020-040710)
Supplement: Supplementary data [file bmjopen-2020-040710supp001.pdf]

Supplementary materials

Seismicity in the Groningen gasfield

Figure S1: Number of earthquakes and volume of gas production in the Groningen gasfield:

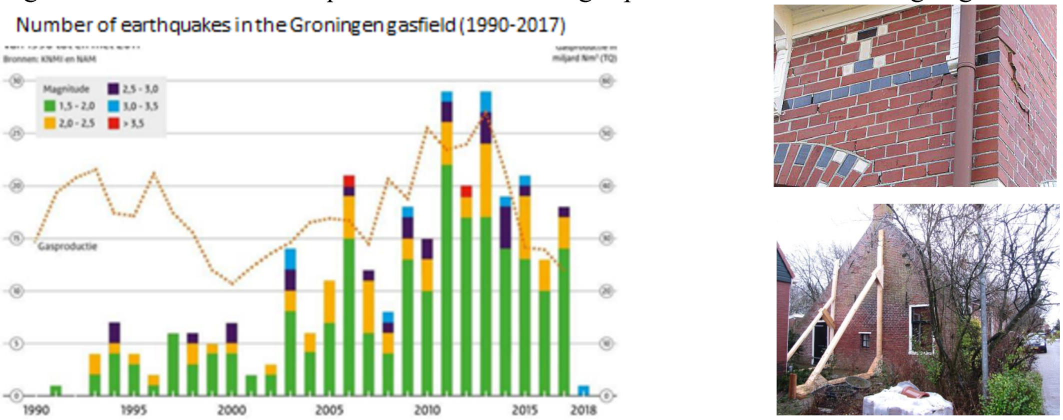

The volume of gas production in the subsoil of Groningen is presented as a dotted line in Figure 2. The number of earthquakes magnitude 2 or higher increased from 2003 onwards. The earthquake of August 2012 in the village of Huizinge (magnitude 3,6 on Richter scale) was a landmark event that caused unrest and great concern among the population in the province of Groningen. It was the heaviest earthquake measured in the province. The magnitude of earthquakes is recorded by the Dutch national borehole network, the regional accelerometer network and all additional seismic stations in the south of the Netherlands. European seismic stations reported the event at epicentral distances up to 800 km (Dost and Kraaipoel, 2012).

410,000 residents of the province of Groningen are exposed to these induced earthquakes: they live in a postcode area where damage has been recognized by the oil company responsible (NAM, a joint venture of Shell and Exxon). Of these, 134,363 adults report having damage to their property (Postmes et al., 2017). Of these 68,343 report having damage multiple times.
Looking worldwide, there are 1174 locations in which induced seismicity is taking place. 11% of seismicity is due to conventional oil or gas extraction (see inducedearthquakes.org/)

References

Dost B and Kraaipoel D. The August 16, 2012 earthquake near Huizinge in Groningen province. Royal Dutch Meteorological Institute, 2013.

Postmes, T., Stroebe, K., Richardson, J., LeKander, B., Oldersma, F., Broer, J. & Greven, F. (2018). Gevolgen van bodembeweging voor Groningers: Ervaren veiligheid, gezondheid en toekomstperspectief 2016-2017. Groningen: Heymans Instituut, Rijksuniversiteit Groningen.

Table S1

Demographic characteristics of participants completing separate measurements per level of damage: total sample size, mean age, distribution of level of education, distribution of level of damage, distribution of gender, and amount of participants that completed the health measures. Netherlands 2016-2017

Damage to house at T1			T1 Feb '16	T2 June '16	T3 Nov '16	T4 Apr '17	T5 Nov '17
None	Total N		1477	1166	968	886	801
	Age (mean)		57.67	59.13	59.13	60.28	61.48
	Level of education (N)	Low	430	340	266	267	238
		Middle	460	351	290	255	226
		High	562	453	396	349	324
	Gender (N)	Male	794	621	515	471	427
		Female	683	545	453	415	374
	Perceived health (N)		1467	-	934	835	784
	Stress related health symptoms (N)		1452	-	937	836	780
	Mental health (N)		1432	1048	920	828	769
One time	Total N		913	730	608	559	490
	Age (mean)		58.32	58.87	58.81	60.06	60.86
	Level of education (N)	Low	237	191	155	143	131
		Middle	295	235	195	182	159
		High	363	295	250	227	193
	Gender (N)	Male	505	398	345	323	279
		Female	407	332	263	236	211
	Perceived health (N)		907	-	587	521	464
	Stress related health symptoms (N)		894	-	584	522	463
	Mental health (N)		895	666	581	517	456
Multiple	Total N		1057	825	704	609	558
	Age (M)		54.06	55.57	55.60	56.71	57.70
	Level of education (N)	Low	215	168	133	120	110
		Middle	381	289	246	213	188
		High	445	356	315	268	253
	Gender (N)	Male	493	385	323	284	269
		Female	563	440	381	325	289
	Perceived health (N)		1048	-	683	578	537
	Stress related health symptoms (N)		1041	-	675	577	530
	Mental health (N)		1018	739	674	570	528

Table S2

Unstandardized regression parameter estimates and standard errors for the association between time, damage, and the interaction between time and damage on perceived health, stress-related health symptoms, and mental health, and the interaction between gender and damage on perceived health, stress-related health symptoms, and mental health – adjusted for gender, age, level of education and ground motion (cumulative PGA). Netherlands 2016-2017.

	Perceived health	Stress-related health symptoms[1]	Mental health
Gender	-0.07	-5.08***	-2.81***
	(0.04)	(0.75)	(0.70)
Age	-0.01***	-0.02	0.07***
	(0.001)	(0.02)	(0.02)
Level of education (middle)	0.08*	0.62	0.99
	(0.03)	(0.67)	(0.62)
Level of education (high)	0.23***	3.03***	2.92***
	(0.03)	(0.63)	(0.59)
Cumulative PGA	-0.001	0.03	-0.01
	(0.004)	(0.09)	(0.08)
Time	-0.01	-0.25*	-0.49***
	(0.01)	(0.13)	(0.15)
Damage (one time)	-0.02	-0.19	-0.05
	(0.04)	(0.93)	(0.81)
Damage (multiple)	-0.14**	-4.04***	-3.79***
	(0.04)	(0.95)	(0.84)
Time * Damage (one time)	-0.02	-0.13	-0.07
	(0.01)	(0.20)	(0.24)
Time * Damage (multiple)	-0.03***	-0.45*	-0.60**
	(0.01)	(0.19)	(0.23)
Gender * Damage (one time)	0.03	-0.58	-0.51
	(0.06)	(1.21)	(1.13)
Gender * Damage (multiple)	0.04	-0.55	0.85
	(0.05)	(1.15)	(1.07)
Constant	3.87***	80.04***	77.86***
	(0.03)	(0.73)	(0.65)
Observations	10,256	9,100	9,686
Log Likelihood	-10,104.34	-36,204.85	-38,019.84
Akaike Inf. Crit.	20,242.68	72,443.70	76,073.68
Bayesian Inf. Crit.	20,365.69	72,564.67	76,195.71

Note. *p<0.05; **p<0.01; ***p<0.001

1. Stress-related health symptoms were reverse-coded such that higher levels indicate less stress
