# Supplementary material for: Chronic disaster impact: the long-term psychological and physical health consequences of housing damage due to induced earthquakes
Source: BMJ Open. 2021 May 5;11(5):e040710. doi: 10.1136/bmjopen-2020-040710 (PMC8103378; doi:10.1136/bmjopen-2020-040710)
Supplement: Author's manuscript [file bmjopen-2020-040710.draft_revisions.pdf]

BMJ Open

BMJ Open is committed to open peer review. As part of this commitment we make the peer review history of every article we publish publicly available.

When an article is published we post the peer reviewers' comments and the authors' responses online. We also post the versions of the paper that were used during peer review. These are the versions that the peer review comments apply to.

The versions of the paper that follow are the versions that were submitted during the peer review process. They are not the versions of record or the final published versions. They should not be cited or distributed as the published version of this manuscript.

BMJ Open is an open access journal and the full, final, typeset and author-corrected version of record of the manuscript is available on our site with no access controls, subscription charges or pay-per-view fees (http://bmjopen.bmj.com).

If you have any questions on BMJ Open's open peer review process please email info.bmjopen@bmj.com

BMJ Open

Long-term psychological and physical health consequences of induced earthquakes: The impact of gas extraction on residents in Groningen

Journal:	*BMJ Open*
Manuscript ID	bmjopen-2020-040710
Article Type:	Original research
Date Submitted by the Author:	20-May-2020
Complete List of Authors:	Stroebe, Katherine; Rijksuniversiteit Groningen, Social Psychology<br>Postmes, Tom; Rijksuniversiteit Groningen, Social Psychology<br>Kanis, Babet; Rijksuniversiteit Groningen, Social Psychology<br>Richardson, Justin; Rijksuniversiteit Groningen, Social Psychology<br>Oldersma, Frans; Department for Statistics and Research, Municipality of Groningen, Groningen, Netherlands<br>Broer, Jan; Municipal Health Services Groningen, ABPG;<br>Greven, Frans; Municipal Health Services Groningen, the Netherlands, Department of Environmental Health
Keywords:	MENTAL HEALTH, EPIDEMIOLOGY, PUBLIC HEALTH

SCHOLARONE™
Manuscripts

I, the Submitting Author has the right to grant and does grant on behalf of all authors of the Work (as defined in the below author licence), an exclusive licence and/or a non-exclusive licence for contributions from authors who are: i) UK Crown employees; ii) where BMJ has agreed a CC-BY licence shall apply, and/or iii) in accordance with the terms applicable for US Federal Government officers or employees acting as part of their official duties; on a worldwide, perpetual, irrevocable, royalty-free basis to BMJ Publishing Group Ltd ("BMJ") its licensees and where the relevant Journal is co-owned by BMJ to the co-owners of the Journal, to publish the Work in this journal and any other BMJ products and to exploit all rights, as set out in our licence.

The Submitting Author accepts and understands that any supply made under these terms is made by BMJ to the Submitting Author unless you are acting as an employee on behalf of your employer or a postgraduate student of an affiliated institution which is paying any applicable article publishing charge ("APC") for Open Access articles. Where the Submitting Author wishes to make the Work available on an Open Access basis (and intends to pay the relevant APC), the terms of reuse of such Open Access shall be governed by a Creative Commons licence – details of these licences and which Creative Commons licence will apply to this Work are set out in our licence referred to above.

Other than as permitted in any relevant BMJ Author's Self Archiving Policies, I confirm this Work has not been accepted for publication elsewhere, is not being considered for publication elsewhere and does not duplicate material already published. I confirm all authors consent to publication of this Work and authorise the granting of this licence.

For peer review only

Long-term psychological and physical health consequences of induced earthquakes: The impact of gas extraction on residents in Groningen

Stroebe, K.[1], Kanis, B.[1], Richardson, J.[1], Oldersma, F.[2], Broer, J.[3], Greven, F.[3] & Postmes, T.[1],

1. University of Groningen, Faculty of Behavioral and Social Sciences, Groningen, Netherlands
2. Department for Statistics and Research, Municipality of Groningen, Groningen, Netherlands
3. Municipal Health Services Groningen, Groningen, Netherlands

Corresponding author: Katherine Stroebe, k.e.stroebe@rug.nl, +313636509

For peer review only - http://bmjopen.bmj.com/site/about/guidelines.xhtml

Abstract

Objectives: The aim was to evaluate the long-term (psychosomatic) health consequences of earthquakes caused by gas extraction compared to a non-exposure control group. Exposure (versus non-exposure) was hypothesized to have a negative impact on (psychosomatic) health outcomes. Impact was expected to increase over time.

Setting — Large scale gas extraction in the Netherlands that induces earthquakes and considerable damage to housing.

Participants — A representative sample of inhabitants randomly selected from municipal population records was contacted 5 times during 21 months (t1:N=3934; t5:N=2156; mean age: 56.54; 50%males; 47%females)

Main (outcome) measures — (Psychosomatic) health outcomes assessed via perceived health, stress related health symptoms, mental health. Independent variable was personal exposure to the consequences of induced earthquakes - assessed via experienced damage to housing (classified into no, once and repeated damages).

Results: Exposure to induced earthquakes has negative health consequences mainly for those whose homes were damaged repeatedly. Compared to a no-damage control group, repeated damage was associated with lower perceived health (OR:1.64), mental health (OR:1.81) and more stress-related health symptoms (OR:2.52). Health effects increased over time: In terms of relative risk, by time 5, those whose homes had repeated damage were 1.61 times more likely to report poor health, 2.08 times more likely to report negative mental health and 2.86 times more at risk of elevated stress related health symptoms.

Conclusion

There are indications that induced earthquakes can pose health risks, but little is known about their long-term impact. The present study is the first to provide evidence that induced earthquakes can have negative health consequences for inhabitants. It identifies which subpopulation is particularly at risk and why. These findings can have important implications for the prevention of negative health consequences of induced earthquakes.

For peer review only - http://bmjopen.bmj.com/site/about/guidelines.xhtml

Keywords: induced earthquakes; seismicity; longitudinal; psychosomatic health, gas extraction

Strengths and limitations

- The long-term impact of induced seismicity is not well documented despite concerns about potential health risks thereof. The present study employs a longitudinal panel design to study the health consequences of those exposed to the consequences of induced earthquakes compared to those not-exposed.
- The present study provides first time evidence that gas extraction that causes induced earthquakes poses severe and long-term (psychosomatic) health risks. It stresses the vulnerability of exposed populations and can provide important input for future decision making, monitoring and contingency planning.
- Younger respondents were somewhat underrepresented in our sample and there was attrition over time. However, attrition was no different for the exposed and non-exposed groups, unrelated to health outcomes, and all further analyses suggest that neither attrition nor sample characteristics had any substantial influence on results and conclusions drawn.

For peer review only

INTRODUCTION

Recent years have seen a rise in induced seismicity due to human activities such as fracking, mining or gas extraction. This development is expected to continue. While smaller in magnitude than natural seismicity, induced seismicity can expose populations to considerable physical (e.g., damage to housing) and social risks (e.g., conflicts between residents and institutions). Moreover, this exposure is recurrent and chronic over time. While there is some insight into the long-term health risks of naturally occurring seismicity, little is known about the impact of induced seismicity. Given the increased use of energy technologies associated with seismicity, also in densely populated areas, knowledge of its health impact is important [1,2].

Naturally occurring seismicity is associated with mental health problems in survivors (e.g., depression, PTSD)[3,4]. These studies are generally cross-sectional and lack an unexposed control group [3]. Moreover, the impact of natural- cannot be equated with that of induced- seismicity for several reasons: Systematic reviews suggest there is *lower* prevalence of mental health impairment for natural compared to human/technological disasters[5,6]; but see [7]. Additionally, different stressors are at play: Natural seismicity can be of greater magnitude, causing death and extensive damages to buildings. For induced seismicity, the maximum magnitude of earthquakes tends to be smaller [8,9]. Risks involve damage to property and an incremental impact on health, as residents are exposed to long-term stressors (e.g., damages; changing community relations; conflicts of interest with powerful institutions [10,11]).

Factual information regarding the health impact of induced seismicity is sparse. Cross-sectional self-report studies([12-14] and an evaluation of health records of

For peer review only - http://bmjopen.bmj.com/site/about/guidelines.xhtml

exposed adults[15] in the context of unconventional gas extraction, suggest associations between induced seismicity and increased (psychosomatic) health symptoms (e.g., sleep disruption, headaches, stress). It is difficult to draw conclusions regarding the impact of seismicity from such studies: Exposure to (the consequences of) seismicity is not distinguished from other risk factors (e.g., wastewater injections). Additionally, most studies lack a non-exposed control group and thus a reliable baseline comparison. None we are aware of consider the longitudinal effects of exposure.

This lack of information regarding the (long-term) impact of induced seismicity on health is problematic. The occurrence of induced earthquakes is increasingly common across the globe, 1174 projects worldwide report induced seismicity [16]. High-profile cases of induced earthquakes have occurred in Oklahoma, U.S.A., and (on a smaller scale) Lancashire, UK [17,18]. There are rising concerns regarding the consequences thereof within exposed populations, coupled with calls to policy makers for monitoring and contingency planning[17]. Policy makers need to weigh the wider economic (and sometimes environmental) benefits against potential drawbacks for exposed residents[19].

The present work was designed to address the lack of information regarding the long-term impact of induced seismicity for residents: It studies the longitudinal (psychosomatic) health impact of induced seismicity on a group exposed to the consequences of seismicity (damage to housing) versus a control group not exposed to these consequences. The present study is novel in charting the chronic impact of exposure to damage on health over a time period of almost two years, on a large sample. We tested the following hypotheses: 1. Exposure versus non-exposure will

For peer review only - http://bmjopen.bmj.com/site/about/guidelines.xhtml

have a negative impact on (psychosomatic) health outcomes. 2. Increases in exposure are related to poorer health outcomes.

METHOD

Setting and exposure

The study was conducted in the province of Groningen, Netherlands, where conventional gas extraction from the largest gas field in Europe takes place. Exposed residents experience rising concerns about physical safety, loss of property value and uncertainty about the future[20,21].The benefits of extraction flow to the operator (the Netherlands' petroleum company) and the national government, while damage repair and compensation by these entities has been criticized as being inadequate [20].

Seismicity has increased over time. While the magnitude of seismic events (up to 3.6 Richter) is generally considered 'light', their impact is felt well beyond the gas field boundaries. Also, multiple factors (limited depth & high rates of occurrence of earthquakes; surface constitution) contribute to considerable damage to housing in a region not prepared for seismic activity [22]. For these reasons, documented damage has proven the most proximal measure of exposure, compared to indicators of seismicity [23].

Sample and recruitment

A stratified random sample was drawn of 25000 residents of the province of Groningen, aged 16 and over, from the official municipal population records which is a complete register of all legal residents. Sampling occurred in areas where damage is reported

For peer review only - http://bmjopen.bmj.com/site/about/guidelines.xhtml

and from outlying areas where this is not the case. Postal-code areas that were rural and strongly affected by damage were oversampled[1]. Participants received letters with personal login codes and one reminder. Eighteen percent signed up. Baseline equivalence of non-exposed groups and exposed groups was assessed. Differences between groups were significant but small. Those with multiple damage to homes were slightly younger (r^2 =.014), more highly educated (Cramer's V=.062), and more likely to be male (V=.072). The first two characteristics suggest the exposed group might be slightly healthier. We statistically controlled for these characteristics.

Data sources

Procedure

Questionnaires were sent via an email link or by post. A reminder was sent after 2 weeks. Participants (t1: N=3934; t5: N=2156) completed measures at 5 time points during 2 years (T1: February 2016, T2: June 2016; T3: October 2016; T4: April 2017; T5: October 2017; see Table 2).

Study Variables

Exposure to consequences of gas extraction was operationalized in two ways. Physical exposure to ground motion was assessed by calculating the cumulative peak ground acceleration (PGA_{cum}) on the basis of "shakemaps" provided by the Dutch geological

[1] In the Netherlands, 4-number postal-code areas provide reasonably accurate geographic positioning, whilst preserving anonymity. Data about damage in each area was provided by the institution handling damage claims, the Centrum voor Veilig Wonen.

For peer review only - http://bmjopen.bmj.com/site/about/guidelines.xhtml

survey (KNMI)[2]. Personal exposure to damage due to gas extraction was assessed by asking participants to indicate how often their home had been damaged (never, once or multiple times)[3].

Demographic variables included gender, age and education level (categorized into 'low', 'middle' or 'high' level of education).

(Psychosomatic) health outcomes were assessed at (almost) all time points (Table 2) as follows via:

1. The WHO and Statistics Netherlands recommended health survey item assessing *self-rated health [24]* ('how good is your health in general?', from 'very poor' to 'excellent' on a 5 point scale), which is part of the SF-36 [25].

2. *Stress-related health symptoms,* based on prior research on symptoms of disaster impact [26]. This list of symptoms was shortened by authors (JB, FG, TP): symptoms associated with chronic stress were retained[4]. Consequences of exposure to toxic substances and noise (e.g., hearing problems) were deemed irrelevant for earthquakes and removed. Ten symptoms (stomach problems, heart palpitations, headaches, dizziness/lightheadedness, sensitivity to light/sounds, muscle/joint pains, irritability, memory/concentration problems, insomnia, tiredness) were assessed by asking 'how often have you experienced the following complaint(s) in the past four weeks' with response options 'never, rarely, occasionally, often, most times, continuously'. Aggregate health index scores were computed for stress-related health

---

[2] KNMI calculates shakemaps based on motion sensor readings. For each participant, the PGA of all events modelled by KNMI between 2012 and 2017 was summed, to create an index of exposure to ground motion before and during the study.
[3] See supplementary materials (table S2) for demographic characteristics by level of damage exposure.
[4] Notably, at the level of individuals who suffer these complaints they are referred to as "medically unexplained" because they can have multiple sources, among which is chronic stress.

For peer review only - http://bmjopen.bmj.com/site/about/guidelines.xhtml

symptoms, so that individuals have a score of 0 to 100, with 100 representing optimal health. Psychometric properties of the aggregate scale were adequate. Correlations among items ranged from ordinal rho 0.26 to 0.72 (median=0.39). A single factor explained 46% of variance. Scale reliability was good with omega=.90.

3. The five item Mental Health Inventory (MHI-5), part of the Short Form Health Survey (SF-36), measuring general *mental health [25,27]*. The MHI-5 has a score of 0 to 100. A score of 100 represents optimal mental health.

Data management and Analysis

Analyses controlled for age, gender and education level. Analyses were weighted to correct for sampling effects of age, gender and degree of exposure of postal-code areas[5]. The weights were developed to counteract any potential distortive effect due to age composition, among others (e.g., because younger people were underrepresented, see results section). We report the weighted results. The unweighted results were very similar.

To assess the impact of exposure to gas extraction on health over time, we constructed multilevel conditional growth models on the three health indices with damage to housing as the (between group) predictor[28]. Participants with missing data on the health indices were retained, as multilevel modelling is robust to missingness in estimation of model outcomes.

[5] As mentioned, we oversampled rural areas as well as the most heavily exposed areas. The geographical weighting was added to control for this overrepresentation.

For peer review only - http://bmjopen.bmj.com/site/about/guidelines.xhtml

Models were tested in a step-wise approach, first including control variables (gender, age, level of education) and time. At the next step, physical exposure (PGA_{cum}) was added, followed by earthquake damage at time 1 and the increase of damage since time 1. The final model included the interaction between damage and time. Model fit was compared to assess which variables best predicted health outcomes. The best fitting models were those including the interaction of damage by time (see Table 3).

To highlight the implications of the findings, we distinguished poor and good health on the basis of health scores, enabling us to compute odds ratios (OR) and relative risk. For mental health we used the conventional criterion of MHI < 60 as cutoff [29]. For perceived health we classified “good” and “outstanding” as good health and all other scale points as poor (conform international convention). For symptoms we devised our own cutoff based on distributional characteristics combined with content criteria: A classification of < 60 as poor health resulted in 9% of the unaffected population being classified as such. Odds ratios were calculated in weighted models, controlling for age, education and gender.

Public involvement

The research setup (design and outcome measures) was discussed with an advisory board consisting of institutions (e.g., local municipalities) and representatives of the public (e.g., action groups). The present work has been disseminated in a public report.

For peer review only - http://bmjopen.bmj.com/site/about/guidelines.xhtml

RESULTS

Sample characteristics

There were no significant fluctuations in sample composition over time in terms of gender, education level and damage to own housing (see Table 2). Young respondents were underrepresented. There was attrition during the study. Dropout characteristics revealed no differences between exposed vs. control groups and no association between dropout and health. Analyses showed no indications that attrition influenced any of the effects reported below. Over time, the average age of participants increased, as young people tended to have a higher likelihood of dropout. It is important to note that additional analyses found no significant interaction effect between age and exposure, suggesting that the effects of exposure were age-independent. Because the sample was not entirely representative and attrition relatively high, we carefully checked the potential consequences thereof and found no indications this influenced results.

Regarding levels of exposure, we know, based on existent data about damage per postal code[6], that the rates of exposure vary substantially within the region: in central areas up to 100% of homes have reported damage at least once. Outside these areas, there is progressively less damage. A substantial part of the province has (nearly) no damage. Average levels of damage are closely associated with ground motion[23]: In postal-code areas where 0% damage was reported until january 2016, there was hardly any exposure to ground motion (total ground motion PGA_{cum}=.07 mm/s^2). Only 3% of the sample located in this area suspected having damage due to earthquakes. In the areas where up to 20% damage was previously reported, ground

[6] Provided by the institution handling damage claims, the Centrum voor Veilig Wonen

For peer review only - http://bmjopen.bmj.com/site/about/guidelines.xhtml

motion levels were somewhat higher PGA_{cum}=0.64 mm/s$^{2)}$) and more people, 26% of the sample, indicated suspecting they have damage. And in the areas where 20% to 100% had reported damage, ground motion was considerably higher, PGA_{cum}=4.13 mm/s^2 and a very high percentage of our sample, 83%, suspected having damage.

The impact of exposure to gas extraction on health over time

The analyses of conditional growth models on self-rated health, stress-related health problems and mental health showed consistent results across all three indicators. Table 3 shows the final results for all variables.

Important to note is that, after including control variables, there was a significant effect of exposure to physical ground motion (PGA_{cum}) on all three health indicators: more ground motion was associated with poorer health. The effect of time was also significant: over time, health deteriorated. In the next step, we included damage to housing. Importantly, the effect of ground motion was suppressed by the larger effects of exposure to multiple damage on all health indicators (p's<.01). This means that damage better predicts health outcomes than ground motion. Having damage once had no significant effect on any of the health indicators. Only participants with multiple damages experienced negative health consequences.

In step 3, the significant 'multiple damage (vs no damage) X time' interaction reveals that exposure to multiple damages is associated with a deterioration of health over time. The inclusion of this interaction variable improved model fit.

To interpret the effects and assess their magnitude, we calculated odds ratios for health measures at every time point, as well as the average impact of exposure over time (Table 4). Inhabitants exposed to damage once are only marginally (and not

For peer review only - http://bmjopen.bmj.com/site/about/guidelines.xhtml

significantly) affected compared with a no-damage control group (odds ratios range from 1.10 to 1.20). Those exposed to damage multiple times are more likely to report poor self-rated health (OR = 1.64, with a 95% confidence interval of 1.31;2.04), more stress-related health symptoms (OR = 2.52 [1.89;3.38]) and less good mental health (OR = 1.81 [1.39;2.37]) than those without damage. This indicates that damage has a considerable impact on participants' health[7].

The table also suggests that differences between groups increase over time. Odds ratios for the difference between those with multiple damage and no damage are considerably higher 21 months after first measurement for self-rated health (OR = 2.00), mental health (OR = 2.32) and stress related health symptoms (OR = 3.36). In terms of relative risk, this means that those whose homes have multiple damage at T5 are 1.61 times more likely to report poor health, 2.08 times more likely to report negative mental health and 2.86 times more at risk of elevated levels of stress related health symptoms.

We also compared the weighted means of the OR's of control variables known to be related to health (age, gender, level of education), in order to further assess the magnitude of the damage effect (Figure 1): How does the effect of damage compare to other known health indicators (e.g., level of education)? Looking at the OR's, you see that they are comparable to known correlates of health such as level of education.

DISCUSSION

Natural and induced seismicity can have negative consequences for local populations due to (acute or accumulated) health threats and irreversible changes to the

[7] We also investigated whether women's health is affected differently by this stressor than men's, but as evidenced in Table S2 in the supplementary materials, this is not the case.

For peer review only - http://bmjopen.bmj.com/site/about/guidelines.xhtml

living environment. Yet, so far studies have not assessed the accumulated impact of (the consequences of) induced seismicity on (psychosomatic) health *over time*. Moreover, most studies lack a non-exposure control group. The present study addresses these shortcomings. Our study provides strong indications that exposure to negative side-effects of induced seismicity (e.g., damage to people's homes) constitutes an increasing health risk over time.

To our knowledge, this is the only study of the long-term impact of induced seismicity on health. Therefore, we can only compare our results with the long-term impacts of very different types of disaster - limiting comparability. For one, the Chernobyl nuclear disaster: Study participants lived in a seriously contaminated area approximately 50 miles from Chernobyl. 6.5 years post disaster, inhabitants were twice as likely to have negative self-rated health (OR:2.25) and psychological distress (OR:1.93), compared to a non-exposed control group[30]. Chernobyl clearly constitutes a very different type of disaster and health risk (radiation exposure). The Brisbane floods were also very different in many respects (e.g., sudden disaster onset; deaths) but with some comparable outcomes, such as considerable damage to homes. 6-7 months post-disaster, those exposed to flooding were twice as likely to report psychological distress compared to the non-exposed[31]. It appears that the health impact of these very different and in many ways more 'acute' disasters are, in terms of effect size, somewhat comparable to the health impact of the more chronic exposure to lower-level seismicity caused by gas extraction. One potential reason for the comparable effect sizes, is that our study focused not just on the environmental effect (e.g., amount of damage in a particular area) but zoomed in on the subgroup who were

For peer review only - http://bmjopen.bmj.com/site/about/guidelines.xhtml

severely affected because they had multiple instances of damage to their own home. We further speculate that the man-made nature of the hazard (the fact that earthquakes are induced) may also enhance the impact on the population.

The present work also provides first time insights into the development of (psychosomatic) health symptoms in response to chronic disaster. In the area of acute disaster response, the few studies on longitudinal health impacts reveal that distress decreases over time[32,33], implying recovery of victims. Our findings suggest that for chronic disasters/hazards, negative effects can accumulate over time, presumably because the recurrent threat leads to an accumulation of stress.

Limitations

Younger respondents were somewhat underrepresented in our sample and there was attrition over time. However, attrition was no different for the exposed and non-exposed groups, unrelated to health outcomes, and all further analyses suggest that neither attrition nor sample characteristics had any substantial influence on results and conclusions drawn above.

Another potential limitation with this type of research is the influence of confounding variables. Yet the following suggests effect sizes are robust: 1. The exposed and control groups were very similar regarding key population and geographical characteristics 2. Follow-up analyses revealed no interactions between any of the population characteristics and the effects of exposure.

One of the three health measures included, stress-related health symptoms, was an adaptation of a previously validated symptoms list (Van de Berg et al., 2005) shortened for this specific study. Although the shortened version was not previously

For peer review only - http://bmjopen.bmj.com/site/about/guidelines.xhtml

validated, it was psychometrically sound. Also, patterns are comparable across health measures, two of which are validated.

One of our exposure measures is self-reported damage. It is possible that damage is perceived differently depending on people's health status. Importantly, physical exposure to ground motion was associated with significant health effects. But effects of damage were stronger. This could be because damage is a more precise and proximate indicator of how individuals are affected by exposure, but also because of recursive effects of (mental) health on perceived damage..

An important issue is generalizability: Is the situation in Groningen comparable to other areas with induced seismicity (e.g., fracking, wastewater injections)? We can only make reasoned inferences. Induced earthquakes are relatively common in energy projects which involve injection[34]. A priori, similar health consequences could occur in all sites in which populations are affected by induced earthquakes. Moreover, the vulnerability of people exposed to seismicity is likely influenced by similar factors: negative consequences are man-made and involve safety, health and social risks [10,11]. In sum, although more research on the impact of induced seismicity is needed [35], we suggest effects are likely to generalize beyond the Groningen case.

Practical implications

The consequences of induced seismicity pose challenges to decision-makers. Benefits to the public good need to be balanced against the welfare of local populations [19]. As projects involving induced seismicity rapidly grow, governments and businesses face decisions whether to invest. Our work provides a case study of what occurs if seismicity is not kept in check. It can increase awareness of the vulnerability of

For peer review only - http://bmjopen.bmj.com/site/about/guidelines.xhtml

exposed populations and provide important input for future decision making, monitoring and contingency planning.

Conclusion

Recent years have seen a rise in induced seismicity. Little is known about the (longitudinal) impact thereof on (psychosomatic) health. The present study is the first to our knowledge evidencing the long-term impact of induced seismicity on health.

For peer review only

Funding: This study was funded by the National Coordinator Groningen.

Contributorship: KS, BK, JR, JB, FO, FG and TP contributed to the research questions and study design. JR and FO contributed to data collection. BK and TP conducted statistical analyses. KS, BK and TP interpreted the results and wrote the initial draft of the manuscript. All authors commented on the final draft of the manuscript. BK and TP had full access to the data in the study and can take responsibility for the integrity of the data and the accuracy of the data analysis.

Conflict of Interest: The authors declare that they have no conflict of interest.

Ethical approval: All procedures performed in studies involving human participants were in accordance with the ethical standards of the ethical board of the department of psychology of the University of Groningen, The Netherlands (research code ppo-015-085).

Informed consent: Informed consent was obtained from all individual participants included in the study.

Data sharing statement: All data collected for this study are not publicly available.

For peer review only

Table 1.
List of definitions

Conventional gas extraction	Extraction through drilling in deep subsoil reservoirs without the injection of chemical liquids.
Fracking	A well stimulation technique in which a rock is fractured by a pressured liquid.
Induced seismicity	Seismic events that are a result of human activity.
Natural seismicity	Seismic events that have a natural cause (e.g., volcanic eruption).
Peak ground acceleration	Measure of the largest increase in ground motion, recorded by a particular station during an earthquake.
Psychosomatic health	Health outcomes involving both mind and body.
Richter scale	Measure of strength of earthquakes with a logarithmic scale.
Shale gas	A natural gas that is trapped in fine grained sediment in rock.
Unconventional gas extraction	Gas reservoirs that require a special stimulation technique to extract gas (e.g., by injecting large quantities of partly chemical fluids underground).

For peer review only

Table 2

Demographic characteristics of participants participating in separate measurements: total number of participants participating in that measurement, decline of number of participants participating as compared to the number of participants participating at T1, mean age, distribution of level of education, distribution of level of damage, distribution of gender, and amount of participants that completed the three health measures in that measurement. Netherlands 2016-2017

		T1<br>Feb '16	T2<br>June '16	T3<br>Nov '16	T4<br>Apr '17	T5<br>Nov '17
Total N		3943	3162	2638	2357	2156
Attrition (compared to T1)		-	19.8%	33.1%	40.2%	45.3%
Age (mean)		56.54	57.75	57.72	58.92	60.00
Level of education (N)	Low	974 (24.7%)	778 (24.6%)	616 (23.4%)	595 (25.2%)	541 (25.1%)
	Middle	1252 (31.8%)	970 (30.7%)	815 (30.9%)	713 (30.3%)	639 (29.6%)
	High	1536 (39.0%)	1241 (39.2%)	1068 (40.5%)	944 (40.1%)	852 (39.5%)
Gender (N)	Male	1970 (50.0%)	1550 (49.0%)	1306 (49.5%)	1185 (50.3%)	1071 (49.7%)
	Female	1855 (47.0%)	1486 (47.0%)	1231 (46.7%)	1100 (46.7%)	993 (46.1%)
Damage to house (N)	None	1483 (37.7%)	1210 (38.3%)	1027 (38.9%)	913 (38.7%)	849 (39.4%)
	One time	916 (23.2%)	626 (19.8%)	554 (21.0%)	505 (21.4%)	459 (21.3%)
	Multiple	1057 (26.8%)	1055 (33.4%)	940 (35.6%)	778 (33.0%)	739 (34.3%)
Perceived health (N)		3830	-	2540	2212	2065
Stress related health symptoms (N)		3776	-	2533	2212	2051
Mental health (N)		3720	2828	2501	2185	2027

For peer review only

Table 3

Unstandardized regression parameter estimates and standard errors for the association between time, damage, and the interaction between time and damage on perceived health, stress-related health symptoms, and mental health – adjusted for sex, age, level of education and ground motion (cumulative PGA). Netherlands 2016-2017.

	Perceived health	Stress-related health symptoms[1]	Mental health
Sex	-0.04	-5.40***	-2.75***
	(0.03)	(0.49)	(0.49)
Age	-0.01***	-0.02	0.07***
	(0.001)	(0.02)	(0.02)
Level of education (middle)	0.07*	0.61	1.30
	(0.03)	(0.67)	(0.67)
Level of education (high)	0.23***	3.02***	3.00***
	(0.03)	(0.63)	(0.63)
Cumulative PGA	0.0001	0.03	-0.03
	(0.004)	(0.09)	(0.08)
Time	-0.01	-0.25*	-0.55***
	(0.01)	(0.13)	(0.15)
Damage (one time)	-0.03	-0.46	-0.40
	(0.04)	(0.75)	(0.67)
Damage (multiple)	-0.15***	-4.31***	-3.70***
	(0.04)	(0.76)	(0.69)
Time * Damage (one time)	-0.01	-0.13	-0.05
	(0.01)	(0.20)	(0.24)
Time * Damage (multiple)	-0.03**	-0.45*	-0.56*
	(0.01)	(0.19)	(0.23)
Constant	3.88***	80.19***	78.12***
	(0.03)	(0.67)	(0.65)
Observations	9,722	9,121	9,180
Log Likelihood	-9,487.06	-36,274.03	-35,912.43
Akaike Inf. Crit.	19,004.11	72,578.06	71,854.87
Bayesian Inf. Crit.	19,111.84	72,684.83	71,961.74

Note. *p<0.05; **p<0.01; ***p<0.001

1. Stress-related health symptoms were reverse-coded such that higher levels indicate less stress

For peer review only

Table 4

Proportion of participants who have poor health and OR of participants who have poor health with damage (compared to those with no damage) across measurements, with 95% confidence intervals – adjusted for age, gender and level of education. Netherlands 2016-2017

Measurement	Damage	Percentage poor health	Odds ratio
Perceived health			
T1	None	22.2% [19.9%;24.5%]	-
	One time	22.5% [19.6%;25.4%]	1.02 [0.82;1.26]
	Multiple	25.6% [22.7%;28.4%]	1.21 [0.99;1.47]
T3	None	21.6% [18.8%;24.3%]	-
	One time	24.4% [20.5%;28.3%]	1.17 [0.90;1.53]
	Multiple	32.4% [29.2%;35.7%]	1.75 [1.41;2.18]
T4	None	21.3% [18.4%;24.2%]	-
	One time	30.0% [25.7%;34.4%]	1.60 [1.22;2.09]
	Multiple	35.5% [31.8%;39.2%]	2.06 [1.63;2.61]
T5	None	23.6% [20.3%;26.9%]	-
	One time	27.5% [22.9%;32.1%]	1.23 [0.92;1.65]
	Multiple	38.0% [34.0%;42.0%]	2.00 [1.57;2.55]
Weighted average	None	22.1% [19.4%;24.9%]	-
	One time	25.6% [21.8%;29.4%]	1.20 [0.93;1.55]
	Multiple	31.8% [28.5%;35.2%]	1.64 [1.31;2.04]
Symptoms			
T1	None	9.2% [7.7%;10.8%]	-
	One time	10.0% [7.9%;12.1%]	1.09 [0.81;1.47]
	Multiple	17.3% [14.9%;19.7%]	2.08 [1.62;2.68]
T3	None	7.1% [5.5%;8.8%]	-
	One time	6.5% [4.3%;8.6%]	0.90 [0.58;1.37]
	Multiple	13.7% 11.4%;16.1%]	2.09 [1.55;2.85]
T4	None	8.1% [6.2%;10.0%	-
	One time	9.4% [6.7%;12.1%]	1.18 [0.78;1.75]
	Multiple	21.8% [18.7%;25.0%]	3.24 [2.40;4.42]
T5	None	7.1% [5.3%;9.0%]	-
	One time	9.1% [6.3%;11.9%]	1.30 [0.84;1.99]
	Multiple	20.3% [17.0;23.5%]	3.36 [2.45;4.68]
Weighted average	None	8.0% [6.3%;9.8%]	-
	One time	8.8% [6.4%;11.2%]	1.10 [0.75;1.60]
	Multiple	18.0% [15.3%;20.7%]	2.52 [1.89;3.38]
Mental health			
T1	None	8.6% [7.1%;10.2%]	
	One time	9.0% [7.0%;11.0%]	1.04 [0.77;1.41]
	Multiple	12.4% [10.3%;14.5%]	1.51 [1.16;1.95]
T2	None	8.7% [7.0%;10.3%]	
	One time	9.2% [6.9%; 11.6%]	1.08 [0.76;1.51]

For peer review only - http://bmjopen.bmj.com/site/about/guidelines.xhtml

	Multiple	18.2% [15.6%;20.7%]	2.37 [1.83;3.07]
T3	None	11.1% [9.0%;13.2%]	
	One time	12.0% [9.2%;14.9%]	1.10 [0.79;1.51]
	Multiple	14.5% [12.1%;16.9%]	1.36 [1.04;1.77]
T4	None	11.8% [9.6%;14.1%]	
	One time	11.8% [8.9%;14.7%]	1.00 [0.71;1.39]
	Multiple	20.2% [17.2%;23.3%]	1.90 [1.46;2.47]
T5	None	9.2% [7.1%;11.4%]	
	One time	12.5% [9.3%;15.7%]	1.40 [0.97;2.02]
	Multiple	19.1% [15.9%;22.2%]	2.32 [1.74;3.12]
Weighted average	None	9.7% [7.9%;11.6%]	-
	One time	10.6% [8.1%;14.2%]	1.10 [0.79;1.53]
	Multiple	16.4% [13.8%;19.0%]	1.81 [1.39;2.37]

Note. Scores were categorised as low health as follows: 1) very poor, poor, or fair perceived health; 2) a score below 60 for stress related health symptoms, and 3) a score below 60 for mental health.

For peer review only

REFERENCES

1 Konkel L. In the Neighborhood of 18 Million: Estimating How Many People Live Near Oil and Gas Wells. *Environ Health Perspect* 2017;**125**:UNSP 124003.

2 U.S. Energy Information Administration. United States Shale gas reserves per state. 2017 Available at: https://www.eia.gov/dnav/ng/ng_enr_shalegas_a_EPG0_R5301_Bcf_a.htm. Accessed 29/01/, 2019.

3 Dai W, Chen L, Lai Z, et al. The incidence of post-traumatic stress disorder among survivors after earthquakes: A systematic review and meta-analysis. *BMC Psychiatry* 2016;**16**.

4 Neria Y, Nandi A, Galea S. Post-traumatic stress disorder following disasters: A systematic review. *Psychol Med* 2008;**38**:467-480.

5 Galea S, Nandi A, Vlahov D. The epidemiology of post-traumatic stress disorder after disasters. *Epidemiol Rev* 2005;**27**:78-91.

6 Norris FH, Friedman MJ, Watson PJ. 60,000 disaster victims speak: Part II. Summary and implications of the disaster mental health research. *Psychiatry: Interpersonal and biological processes* 2002;**65**:240-260.

7 Rubonis AV, Bickman L. Psychological impairment in the wake of disaster: The disaster–psychopathology relationship. *Psychol Bull* 1991;**109**:384.

8 Keranen KM, Weingarten M. Induced Seismicity. *Annual Review of Earth and Planetary Sciences, Vol 46* 2018;**46**:149-174.

9 van der Elst, Nicholas J., Page MT, Weiser DA, et al. Induced earthquake magnitudes are as large as (statistically) expected. *Journal of Geophysical Research-Solid Earth* 2016;**121**:4575-4590.

10 Couch SR, Kroll-Smith JS. The chronic technical disaster: Toward a social scientific perspective. *Social Science Quarterly* 1985;**66**:564.

11 Sangaramoorthy T, Jamison AM, Boyle MD, et al. Place-based perceptions of the impacts of

For peer review only

fracking along the Marcellus Shale. *Soc Sci Med* 2016;**151**:27-37.

12 Rabinowitz PM, Slizovskiy IB, Lamers V, et al. Proximity to Natural Gas Wells and Reported Health Status: Results of a Household Survey in Washington County, Pennsylvania. *Environ Health Perspect* 2015;**123**:21-26.

13 Steinzor N, Subra W, Sumi L. Investigating links between shale gas development and health impacts through a community survey project in Pennsylvania. *New solutions : a journal of environmental and occupational health policy : NS* 2013;**23**:55-83.

14 Tustin AW, Hirsch AG, Rasmussen SG, et al. Associations between Unconventional Natural Gas Development and Nasal and Sinus, Migraine Headache, and Fatigue Symptoms in Pennsylvania. *Environ Health Perspect* 2017;**125**:189-197.

15 Weinberger B, Greiner LH, Walleigh L, et al. Health symptoms in residents living near shale gas activity: A retrospective record review from the Environmental Health Project. *Preventive medicine reports* 2017;**8**:112-115.

16 Wilson MP, Foulger GR, Gluyas JG, et al. HiQuake: The human-induced earthquake database. *Seismol Res Lett* 2017;**88**:1560-1565.

17 Aczel MR, Makuch KE. Shale, Quakes, and High Stakes: Regulating Fracking-Induced Seismicity in Oklahoma, USA and Lancashire, UK. *Case Studies in the Environment* 2019;.

18 Keranen KM, Weingarten M, Abers GA, et al. Sharp increase in central Oklahoma seismicity since 2008 induced by massive wastewater injection. *Science* 2014;**345**:448-451.

19 Fleming RC, Reins L. Shale gas extraction, precaution and prevention: A conversation on regulatory responses. *Energy Research & Social Science* 2016;**20**:131-141.

20 Dutch Safety Board. Earthquake risks in Groningen. Research concerning the role population safety played in decision making procedures about gas extraction (1959-2014) . 2015;.

21 Postmes, T., Stroebe, K., Richardson, J., LeKander, B., Oldersma, F., Broer, J. & Greven, F. Consequences of ground motion for the Groningen population: Experienced safety, health and

For peer review only - http://bmjopen.bmj.com/site/about/guidelines.xhtml

future perspective 2016-2017. *University of Groningen* 2018;.

22 Vlek C. Rise and reduction of induced earthquakes in the Groningen gas field, 1991-2018: statistical trends, social impacts, and policy change. *Environ Earth Sci* 2019;**78**:59.

23 Geurts C, Steenbergen R. Note for the Dutch State Supervision of Mines: The relation between Peak Ground Acceleration and the risk of damage for induced earthquakes in Groningen. *The Netherlands Organisation for Applied Scientific Research* 2016;.

24 Pappas G. Health interview surveys: Towards international harmonization of methods and instruments - deBruin,A, Picavet,HSJ, Nossikoy,A. *Soc Sci Med* 1997;**44**:1431-1432.

25 Ware JE, Gandek B, IQOLA Project. Overview of the SF-36 Health Survey and the International Quality of Life Assessment (IQOLA) Project. *J Clin Epidemiol* 1998;**51**:903-912.

26 Van den Berg B, Yzermans CJ, Van der Velden, Peter G., et al. Risk Factors for Unexplained Symptoms After a Disaster: A Five-Year Longitudinal Study in General Practice. *Psychosomatics* 2009;**50**:69-77.

27 Berwick DM, Murphy JM, Goldman PA, et al. Performance of a five-item mental health screening test. *Med Care* 1991;.

28 Raudenbush SW, Bryk AS. Hierarchical linear models: Applications and data analysis methods. : Sage 2002.

29 Rumpf H, Meyer C, Hapke U, et al. Screening for mental health: validity of the MHI-5 using DSM-IV Axis I psychiatric disorders as gold standard. *Psychiatry Res* 2001;**105**:243-253.

30 Havenaar J, Rumyantzeva G, Kasyanenko A, et al. Health effects of the Chernobyl disaster: Illness or illness behavior? A comparative general health survey in two former Soviet regions. *Environ Health Perspect* 1997;**105**:1533-1537.

31 Alderman K, Turner LR, Tong S. Assessment of the Health Impacts of the 2011 Summer Floods in Brisbane. *Dis Med Public Health Prep* 2013;**7**:380-386.

32 Lowell A, Suarez-Jimenez B, Helpman L, et al. 9/11-related PTSD among highly exposed populations: a systematic review 15 years after the attack. *Psychol Med* 2018;**48**:537-553.

For peer review only

33 van der Velden, Peter G., Wong A, Boshuizen HC, et al. Persistent mental health disturbances during the 10years after a disaster: Four-wave longitudinal comparative study. *Psychiatry Clin Neurosci* 2013;**67**:110-118.

34 Ellsworth WL. Injection-induced earthquakes. *Science* 2013;**341**:1225942.

35 Hirsch JK, Smalley KB, Selby-Nelson EM, et al. Psychosocial Impact of Fracking: a Review of the Literature on the Mental Health Consequences of Hydraulic Fracturing. *International Journal of Mental Health and Addiction* 2018;**16**:1-15.

For peer review only

Figure 1. Weighted average odds ratios.

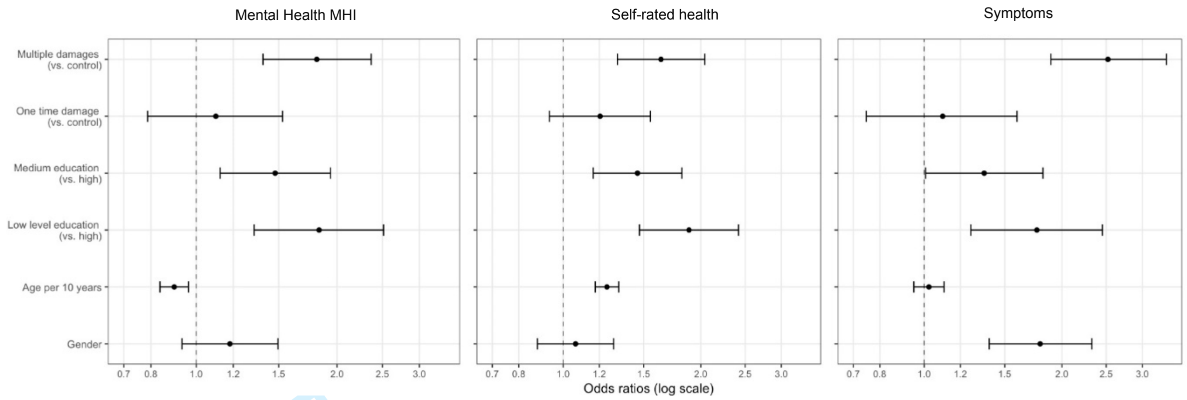

For peer review only

Supplementary materials

Table S1

Demographic characteristics of participants completing separate measurements per level of damage: total sample size, mean age, distribution of level of education, distribution of level of damage, distribution of gender, and amount of participants that completed the health measures. Netherlands 2016-2017

Damage to house at T1			T1 Feb '16	T2 June '16	T3 Nov '16	T4 Apr '17	T5 Nov '17
None	Total N		1483	1172	968	889	804
	Age (mean)		57.66	59.11	59.13	60.28	61.48
	Level of education (N)	Low	433 (29.2%)	343 (29.3%)	266 (27.5%)	270 (30.4%)	241 (30.0%)
		Middle	460 (31.0%)	351 (29.9%)	290 (30.0%)	255 (26.7%)	226 (28.1%)
		High	565 (38.1%)	456 (38.9%)	396 (40.9%)	349 (39.3%)	324 (40.3%)
	Gender (N)	Male	794 (53.5%)	621 (53.0%)	515 (53.2%)	471 (53.0%)	427 (53.1%)
		Female	689 (46.5%)	551 (47.0%)	453 (46.8%)	418 (47.0%)	377 (46.9%)
	Perceived health (N)		1473	-	934	838	787
	Stress related health symptoms (N)		1458	-	937	839	783
	Mental health (N)		1438	1054	920	831	772
One time	Total N		916	733	608	562	493
	Age (mean)		58.36	58.92	58.81	60.11	60.91
	Level of education (N)	Low	240 (26.2%)	194 (26.5%)	155 (25.5%)	146 (26.0%)	134 (27.2%)
		Middle	295 (32.2%)	235 (32.1%)	195 (32.1%)	182 (32.4%)	159 (32.3%)
		High	363 (39.6%)	295 (40.2%)	250 (41.1%)	227 (40.4%)	193 (39.1%)
	Gender (N)	Male	508 (55.5%)	401 (54.7%)	345 (56.7%)	326 (58.0%)	282 (57.2%)
		Female	407 (44.4%)	332 (45.3%)	263 (43.3%)	236 (42.0%)	211 (42.8%)
	Perceived health (N)		910	-	587	524	467
	Stress related health symptoms (N)		897	-	584	525	466
	Mental health (N)		898	669	581	520	459
Multiple	Total N		1057	825	704	609	558
	Age (M)		54.06	55.57	55.60	56.71	57.70
	Level of education (N)	Low	215 (20.3%)	168 (20.4%)	133 (18.9%)	120 (19.7%)	110 (19.7%)
		Middle	381 (36.0%)	289 (35.0%)	246 (34.9%)	213 (35.0%)	188 (33.8%)
		High	445 (42.1%)	356 (43.1%)	315 (44.7%)	268 (44.0%)	253 (45.4%)
	Gender (N)	Male	493 (46.6%)	385 (46.6%)	323 (45.9%)	284 (46.7%)	269 (48.3%)

For peer review only

	Female	563 (53.3%)	440 (53.3%)	381 (54.1%)	325 (53.4%)	289 (51.9%)
Perceived health (N)		1048	-	683	578	537
Stress related health symptoms (N)		1041	-	675	577	530
Mental health (N)		1018	739	674	570	528

For peer review only

Table S2

Unstandardized regression parameter estimates and standard errors for the association between time, damage, and the interaction between time and damage on perceived health, stress-related health symptoms, and mental health, and the interaction between gender and damage on perceived health, stress-related health symptoms, and mental health – adjusted for sex, age, level of education and ground motion (cumulative PGA). Netherlands 2016-2017.

	Perceived health	Stress-related health symptoms[1]	Mental health
Sex	-0.06	-5.30***	-2.97***
	(0.04)	(0.79)	(0.75)
Age	-0.01***	-0.02	0.06***
	(0.001)	(0.02)	(0.02)
Level of education (middle)	0.07*	0.79	1.28
	(0.03)	(0.71)	(0.67)
Level of education (high)	0.23***	3.08***	2.97***
	(0.03)	(0.66)	(0.63)
Cumulative PGA	0.0001	0.003	-0.03
	(0.004)	(0.09)	(0.08)
Time	-0.01	-0.30*	-0.55***
	(0.01)	(0.13)	(0.15)
Damage (one time)	-0.04	-0.35	-0.34
	(0.05)	(0.99)	(0.86)
Damage (multiple)	-0.17***	-4.57***	-4.14***
	(0.05)	(1.02)	(0.90)
Time * Damage (one time)	-0.01	-0.12	-0.05
	(0.01)	(0.21)	(0.24)
Time * Damage (multiple)	-0.03**	-0.38	-0.56*
	(0.01)	(0.20)	(0.23)
Gender * Damage (one time)	0.02	-0.19	-0.16
	(0.06)	(1.27)	(1.19)
Gender * Damage (multiple)	0.03	-0.23	0.86
	(0.06)	(1.22)	(1.15)
Constant	3.88***	80.39***	78.25***
	(0.04)	(0.78)	(0.71)
Observations	9,722	8,588	9,180
Log Likelihood	-9,486.91	-33,969.29	-35,912.03
Akaike Inf. Crit.	19,007.82	67,972.57	71,858.07
Bayesian Inf. Crit.	19,129.92	68,092.56	71,979.19

Note. *p<0.05; **p<0.01; ***p<0.001

1. Stress-related health symptoms were reverse-coded such that higher levels indicate less stress

STROBE Statement—checklist of items that should be included in reports of observational studies

	Item No.	Recommendation	Page No.	Relevant text from manuscript
**Title and abstract**	1	(*a*) Indicate the study's design with a commonly used term in the title or the abstract	1/2	Title is mentioned on page 1
		(*b*) Provide in the abstract an informative and balanced summary of what was done and what was found	2	
**Introduction**				
Background/rationale	2	Explain the scientific background and rationale for the investigation being reported	4/5	
Objectives	3	State specific objectives, including any prespecified hypotheses	5	"The present work was designed to address the lack of information regarding the long-term impact of induced seismicity for residents: It studies the longitudinal (psychosomatic) health impact of induced seismicity on a group exposed to the consequences of seismicity (damage to housing) versus a control group not exposed to these consequences. The present study is novel in charting the chronic impact of exposure to damage on health over a time period of almost two years, on a large sample. We tested the following hypotheses: 1. Exposure versus non-exposure will have a negative impact on (psychosomatic) health outcomes. 2. Increases in

For peer review only - http://bmjopen.bmj.com/site/about/guidelines.xhtml

				exposure are related to poorer health outcomes.
**Methods**				
Study design	4	Present key elements of study design early in the paper	6-9	
Setting	5	Describe the setting, locations, and relevant dates, including periods of recruitment, exposure, follow-up, and data collection	7	. Participants (t1: N=3934; t5: N=2156) completed measures at 5 time points during 2 years (T1: February 2016, T2: June 2016; T3: October 2016; T4: April 2017; T5: October 2017; see Table 2
Participants	6	(*a*) *Cohort study*—Give the eligibility criteria, and the sources and methods of selection of participants. Describe methods of follow-up<br>*Case-control study*—Give the eligibility criteria, and the sources and methods of case ascertainment and control selection. Give the rationale for the choice of cases and controls<br>*Cross-sectional study*—Give the eligibility criteria, and the sources and methods of selection of participants	7	
		(*b*) *Cohort study*—For matched studies, give matching criteria and number of exposed and unexposed<br>*Case-control study*—For matched studies, give matching criteria and the number of controls per case		
Variables	7	Clearly define all outcomes, exposures, predictors, potential confounders, and effect modifiers. Give diagnostic criteria, if applicable	7-9	
Data sources/ measurement	8*	For each variable of interest, give sources of data and details of methods of assessment (measurement). Describe comparability of assessment methods if there is more than one group	7-9	
Bias	9	Describe any efforts to address potential sources of bias	9	
Study size	10	Explain how the study size was arrived at	6-7	

Continued on next page

For peer review only

Quantitative variables	11	Explain how quantitative variables were handled in the analyses. If applicable, describe which groupings were chosen and why	9-10	
Statistical methods	12	(*a*) Describe all statistical methods, including those used to control for confounding	9-10	
		(*b*) Describe any methods used to examine subgroups and interactions	9-10	
		(*c*) Explain how missing data were addressed	9	Participants with missing data on the health indices were retained, as multilevel modelling is robust to missingness in estimation of model outcomes. See Table 2 for an overview of the number of participants completing each health measure per time point.
		(*d*) *Cohort study*—If applicable, explain how loss to follow-up was addressed<br>*Case-control study*—If applicable, explain how matching of cases and controls was addressed<br>*Cross-sectional study*—If applicable, describe analytical methods taking account of sampling strategy	11	we conducted analyses to test whether loss to follow up affected the nature of the results. As we outline on page 11, this was not the case.
		(*e*) Describe any sensitivity analyses		
**Results**				
Participants	13*	(a) Report numbers of individuals at each stage of study—eg numbers potentially eligible, examined for eligibility, confirmed eligible, included in the study, completing follow-up, and analysed	20	Table 2
		(b) Give reasons for non-participation at each stage	N/A	
		(c) Consider use of a flow diagram	N/A	
Descriptive data	14*	(a) Give characteristics of study participants (eg demographic, clinical, social) and information on exposures and potential confounders	20	Table 2
		(b) Indicate number of participants with missing data for each variable of interest	N/A	
		(c) *Cohort study*—Summarise follow-up time (eg, average and total amount)		
Outcome data	15*	*Cohort study*—Report numbers of outcome events or summary measures over time		
		Case-control study—Report numbers in each exposure category, or summary measures of exposure		
		Cross-sectional study—Report numbers of outcome events or summary measures		

For peer review only - http://bmjopen.bmj.com/site/about/guidelines.xhtml

Main results	16	(*a*) Give unadjusted estimates and, if applicable, confounder-adjusted estimates and their precision (eg, 95% confidence interval). Make clear which confounders were adjusted for and why they were included	12-13 &21/22	Table 3/4
		(*b*) Report category boundaries when continuous variables were categorized	12-13& 21/11	Tables 3/4
		(*c*) If relevant, consider translating estimates of relative risk into absolute risk for a meaningful time period		

Continued on next page

For peer review only

Other analyses	17	Report other analyses done—eg analyses of subgroups and interactions, and sensitivity analyses	
**Discussion**			
Key results	18	Summarise key results with reference to study objectives	10-11
Limitations	19	Discuss limitations of the study, taking into account sources of potential bias or imprecision. Discuss both direction and magnitude of any potential bias	12-13
Interpretation	20	Give a cautious overall interpretation of results considering objectives, limitations, multiplicity of analyses, results from similar studies, and other relevant evidence	10-13
Generalisability	21	Discuss the generalisability (external validity) of the study results	12-13
**Other information**			
Funding	22	Give the source of funding and the role of the funders for the present study and, if applicable, for the original study on which the present article is based	24

*Give information separately for cases and controls in case-control studies and, if applicable, for exposed and unexposed groups in cohort and cross-sectional studies.

Note: An Explanation and Elaboration article discusses each checklist item and gives methodological background and published examples of transparent reporting. The STROBE checklist is best used in conjunction with this article (freely available on the Web sites of PLoS Medicine at http://www.plosmedicine.org/, Annals of Internal Medicine at http://www.annals.org/, and Epidemiology at http://www.epidem.com/). Information on the STROBE Initiative is available at www.strobe-statement.org.

For peer review only

BMJ Open

Chronic disaster impact: the long-term psychological and physical health consequences of housing damage due to induced earthquakes

Journal:	*BMJ Open*
Manuscript ID	bmjopen-2020-040710.R1
Article Type:	Original research
Date Submitted by the Author:	04-Jan-2021
Complete List of Authors:	Stroebe, Katherine; Rijksuniversiteit Groningen, Social Psychology<br>Kanis, Babet; Rijksuniversiteit Groningen, Social Psychology<br>Richardson, Justin; Rijksuniversiteit Groningen, Social Psychology<br>Oldersma, Frans; Department for Statistics and Research, Municipality of Groningen, Groningen, Netherlands<br>Broer, Jan; Municipal Health Services Groningen, ABPG;<br>Greven, Frans; Municipal Health Services Groningen, the Netherlands, Department of Environmental Health<br>Postmes, Tom; Rijksuniversiteit Groningen, Social Psychology
**Primary Subject Heading**:	Public health
Secondary Subject Heading:	Mental health, Public health
Keywords:	MENTAL HEALTH, EPIDEMIOLOGY, PUBLIC HEALTH

SCHOLARONE™ Manuscripts

I, the Submitting Author has the right to grant and does grant on behalf of all authors of the Work (as defined in the below author licence), an exclusive licence and/or a non-exclusive licence for contributions from authors who are: i) UK Crown employees; ii) where BMJ has agreed a CC-BY licence shall apply, and/or iii) in accordance with the terms applicable for US Federal Government officers or employees acting as part of their official duties; on a worldwide, perpetual, irrevocable, royalty-free basis to BMJ Publishing Group Ltd ("BMJ") its licensees and where the relevant Journal is co-owned by BMJ to the co-owners of the Journal, to publish the Work in this journal and any other BMJ products and to exploit all rights, as set out in our licence.

The Submitting Author accepts and understands that any supply made under these terms is made by BMJ to the Submitting Author unless you are acting as an employee on behalf of your employer or a postgraduate student of an affiliated institution which is paying any applicable article publishing charge ("APC") for Open Access articles. Where the Submitting Author wishes to make the Work available on an Open Access basis (and intends to pay the relevant APC), the terms of reuse of such Open Access shall be governed by a Creative Commons licence – details of these licences and which Creative Commons licence will apply to this Work are set out in our licence referred to above.

Other than as permitted in any relevant BMJ Author's Self Archiving Policies, I confirm this Work has not been accepted for publication elsewhere, is not being considered for publication elsewhere and does not duplicate material already published. I confirm all authors consent to publication of this Work and authorise the granting of this licence.

For peer review only

Chronic disaster impact: the long-term psychological and physical health consequences of housing damage due to induced earthquakes

Stroebe, K.[1], Kanis, B.[1], Richardson, J.[1], Oldersma, F.[2], Broer, J.[3], Greven, F.[3] & Postmes, T.[1],

1. University of Groningen, Faculty of Behavioral and Social Sciences, Groningen, Netherlands
2. Department for Statistics and Research, Municipality of Groningen, Groningen, Netherlands
3. Municipal Health Services Groningen, Groningen, Netherlands

Corresponding author: Katherine Stroebe, k.e.stroebe@rug.nl, +313636509

For peer review only

Abstract

Objectives: To evaluate the long-term (psychosomatic) health consequences of man-made earthquakes compared to a non-exposure control group. Exposure was hypothesized to have an increasingly negative impact on health outcomes over time.

Setting — Large scale gas extraction in the Netherlands causing earthquakes and considerable damage.

Participants — A representative sample of inhabitants randomly selected from municipal population records; contacted 5 times during 21 months (T1:N=3934; T5:N=2150; mean age: 56.54; 50%males; At T5, N=846 (39.3%) had no, 459 (21.3%) once, and 736 (34.2%) repeated damages.

Main measures — (Psychosomatic) health outcomes: Self rated health and Mental Health Inventory (both: validated; Short Form Health Survey); stress related health symptoms (shortened version of previously validated symptoms list) Independent variable: Exposure to the consequences of earthquakes assessed via physical (Peak Ground Acceleration, PGA) and personal exposure (damage to housing: none, once, repeated).

Results: Exposure to induced earthquakes has negative health consequences mainly for those whose homes were damaged repeatedly. Compared to a no-damage control group, repeated damage was associated with lower self-rated health (OR:1.64), mental health (OR:1.83) and more stress-related health symptoms (OR:2.52). Effects increased over time: In terms of relative risk, by T5, those whose homes had repeated damage were respectively 1.60 and 2.11 times more likely to report poor health and negative mental health and 2.84 times more at risk of elevated stress related health symptoms. Results for physical exposure were comparable.

Conclusion

This is the first study to provide evidence that induced earthquakes can have negative health consequences for inhabitants over time. It identifies the subpopulation particularly at risk: people with repeated damages/high PGA). Findings can have important implications for the prevention of negative health consequences of induced earthquakes.

2

For peer review only

Keywords: induced earthquakes; seismicity; longitudinal; psychosomatic health, gas extraction

Strengths and limitations

- The present study employs a longitudinal panel design with 5 measurement points to study health consequences of gas extraction
- The study has an exposed (residents with damage to housing) and a non-exposed (residents with no damage) control group
- Two health measures (self-rated health; Mental Health Inventory) were previously validated, the third was an adaptation of a previously validated symptoms list
- Younger respondents were somewhat underrepresented in our sample
- There was 45.3% attrition over time but attrition was no different for the exposed versus non-exposed groups and was unrelated to health outcomes.

For peer review only

INTRODUCTION

Recent years have seen a rise in induced seismicity due to human activities such as fracking, mining or gas extraction. This development is expected to continue. While smaller in magnitude than natural seismicity, induced seismicity can expose populations to considerable physical (e.g., damage to housing) and social risks (e.g., conflicts between residents and institutions). Moreover, this exposure is recurrent and chronic over time. While there is some insight into the long-term health risks of naturally occurring seismicity, little is known about the impact of induced seismicity. Given the increased use of energy technologies associated with seismicity, also in densely populated areas, knowledge of its health impact is important [1, 2] (see also Table 1 for definitions of gas-extraction related terminology).

Naturally occurring seismicity is associated with mental health problems in survivors (e.g., depression, PTSD)[3-5]. While there has been some increase in studies considering longitudinal health effects of seismicity, lack of longitudinal design and an unexposed control group have been highlighted as major concerns for studies of natural disasters [3, 5, 6]. Moreover, the impact of natural- cannot be equated with that of induced- seismicity for several reasons: Systematic reviews suggest there is *lower* prevalence of mental health impairment for natural compared to human/technological disasters [7, 8]; but see [9]. Additionally, different stressors are at play: Natural seismicity can be of greater magnitude, causing death and extensive damages to buildings. For induced seismicity, the maximum magnitude of earthquakes tends to be smaller [10, 11]. Risks involve damage to property and an incremental impact on health,

For peer review only

For peer review only - http://bmjopen.bmj.com/site/about/guidelines.xhtml

as residents are exposed to long-term stressors (e.g., damages; changing community relations; conflicts of interest with powerful institutions [12, 13]).

Factual information regarding the health impact of induced seismicity is sparse. Cross-sectional self-report studies [14-16] and an evaluation of health records of exposed adults[17] in the context of unconventional gas extraction, suggest associations between induced seismicity and increased (psychosomatic) health symptoms (e.g., sleep disruption, headaches, stress). It is difficult to draw conclusions regarding the impact of seismicity from such studies: Exposure to (the consequences of) seismicity is not distinguished from other risk factors (e.g., wastewater injections). Additionally, most studies lack a non-exposed control group and thus a reliable baseline comparison. None we are aware of consider the longitudinal effects of exposure.

This lack of information regarding the (long-term) impact of induced seismicity on health is problematic. The occurrence of induced earthquakes is increasingly common across the globe, 1174 projects worldwide report induced seismicity [18]. High-profile cases of induced earthquakes have occurred in Oklahoma, U.S.A., and (on a smaller scale) Lancashire, UK [19, 20]. There are rising concerns regarding the consequences thereof within exposed populations, coupled with calls to policy makers for monitoring and contingency planning [19]. Policy makers need to weigh the wider economic (and sometimes environmental) benefits against potential drawbacks for exposed residents [21].

The present work was designed to address the lack of information regarding the long-term impact of induced seismicity for residents: It studies the longitudinal (psychosomatic) health impact of induced seismicity on a group exposed to the

For peer review only - http://bmjopen.bmj.com/site/about/guidelines.xhtml

consequences of seismicity (damage to housing) versus a control group not exposed to these consequences. The study was conducted in the province of Groningen, Netherlands, where conventional gas extraction from the largest gas field in Europe takes place[1]. While the magnitude of seismic events (up to 3.6 Richter) is generally considered 'light', their magnitude has increased over the past 30 years, making this a chronic disaster, and their impact is felt well beyond the gas field boundaries. The earthquakes cause considerable damage to housing in a region not prepared for seismic activity [22] and governmental responses to damage compensation have been considered inadequate [23]

The present study is novel in charting the chronic impact of exposure to damage on health over a time period of almost two years, on a large sample. We tested the following hypotheses: 1. Exposure versus non-exposure will have a negative impact on (psychosomatic) health outcomes. 2. Increases in exposure are related to poorer health outcomes.

METHOD

Setting and exposure

The study was conducted in the province of Groningen, Netherlands, where conventional gas extraction from the largest gas field in Europe takes place. Exposed residents experience rising concerns about physical safety, loss of property value and uncertainty about the future[23, 24].The benefits of extraction flow to the operator (the

[1] See Figure S1 for more information on seismicity in this province

For peer review only - http://bmjopen.bmj.com/site/about/guidelines.xhtml

Netherlands' petroleum company) and the national government, while damage repair and compensation by these entities has been criticized as being inadequate[23].

Seismicity has increased over time. While the magnitude of seismic events (up to 3.6 Richter) is generally considered 'light', their impact is felt well beyond the gas field boundaries. Also, multiple factors (limited depth & high rates of occurrence of earthquakes; surface constitution) contribute to considerable damage to housing in a region not prepared for seismic activity[22]. For these reasons, documented damage has proven the most proximal measure of exposure, compared to indicators of seismicity[25].

Sample and recruitment

A stratified random sample was drawn of 25000 residents of the province of Groningen, aged 16 and over, from the official municipal population records which is a complete register of all legal residents. Sampling occurred in areas where damage is reported and from outlying areas where this is not the case. Postal-code areas that were rural and strongly affected by damage were oversampled[2]. Residents received letters with personal login codes and one reminder. Eighteen percent (N=4577) signed up for the study, and later received invitations to all questionnaires. Of these 4577, 86% (3934) filled out the first questionnaire. Baseline equivalence of non-exposed and exposed groups was assessed. Differences between groups were significant but small. Those with multiple damage to homes were slightly younger (r^2 =.014), more highly educated

[2] In the Netherlands, 4-number postal-code areas provide reasonably accurate geographic positioning, whilst preserving anonymity. Data about damage in each area was provided by the institution handling damage claims, the Centrum voor Veilig Wonen.

For peer review only - http://bmjopen.bmj.com/site/about/guidelines.xhtml

(Cramer's V=.062), and more likely to be male (V=.072). The first two characteristics suggest the exposed group might be slightly healthier. We statistically controlled for these characteristics.

Data sources

Procedure

Questionnaires were sent via an email link or by post. A reminder was sent after 2 weeks. Participants (T1: N=3934; T5: N=2150) completed measures at 5 time points during 2 years (T1: February 2016, T2: June 2016; T3: November 2016; T4: April 2017; T5: November 2017; see Table 2).

Study Variables

Exposure to consequences of gas extraction was operationalized in two ways. Physical exposure to ground motion was assessed by calculating the cumulative peak ground acceleration (PGA_{cum}) on the basis of "shakemaps" provided by the Dutch geological survey (KNMI)[3]. Personal exposure to damage due to gas extraction was assessed every timepoint by asking participants to indicate how often their home had been damaged (never, once or multiple times)[4].

[3] KNMI calculates shakemaps based on motion sensor readings. For each participant, the PGA of all events modelled by KNMI between 2012 and 2017 was summed, to create an index of exposure to ground motion before and during the study.
[4] See supplementary materials (table S1) for demographic characteristics by level of damage exposure.

For peer review only - http://bmjopen.bmj.com/site/about/guidelines.xhtml

Demographic variables included gender, age and completed education level (categorized into ‘low’ (no, elementary, or pre-vocational education), ‘middle’ (secondary or vocational education), or ‘high’ (higher education) level of education).

(Psychosomatic) health outcomes were assessed at (almost) all time points (Table 2) as follows via:

1. The WHO and Statistics Netherlands recommended validated health survey item assessing *self-rated health [26]* (‘how good is your health in general?’, from ‘very poor’ to ‘excellent’ on a 5 point scale), which is part of the SF-36 [27].

2. *Stress-related health* symptoms were *based on a validated scale of* symptoms of disaster impact [28]. This list of symptoms was shortened by authors (JB, FG, TP): symptoms associated with chronic stress were retained[5]. Consequences of exposure to toxic substances and noise (e.g., hearing problems) were deemed irrelevant for earthquakes and removed. Ten symptoms (stomach problems, heart palpitations, headaches, dizziness/lightheadedness, sensitivity to light/sounds, muscle/joint pains, irritability, memory/concentration problems, insomnia, tiredness) were assessed by asking ‘how often have you experienced the following complaint(s) in the past four weeks’ with response options ‘never, rarely, occasionally, often, most times, continuously’. Aggregate health index scores were computed for stress-related health symptoms, so that individuals have a score of 0 to 100, with 100 representing optimal health. Psychometric properties of the aggregate scale were adequate. Correlations among items ranged from ordinal rho 0.26 to 0.72 (median=0.39). A single factor explained 46% of variance. Scale reliability was good with omega=.90.

[5] Notably, at the level of individuals who suffer these complaints they are referred to as “medically unexplained” because they can have multiple sources, among which is chronic stress.

For peer review only - http://bmjopen.bmj.com/site/about/guidelines.xhtml

3. The five-item validated Mental Health Inventory (MHI-5), part of the Short Form Health Survey (SF-36), measuring general *mental health [27, 29]*. The MHI-5 has a score of 0 to 100. A score of 100 represents optimal mental health.

Data management and Analysis

Analyses controlled for age, gender and education level. Analyses were weighted to correct for sampling effects of age, gender and degree of exposure of postal-code areas[6]. The weights were developed to counteract any potential distortive effect due to age composition, among others (e.g., because younger people were underrepresented, see results section). We report the weighted results. The unweighted results were very similar.

To assess the impact of exposure to gas extraction on health over time, we constructed multilevel conditional growth models on the three health indices with damage to housing as the (between group) predictor[30]. Participants with missing data on the health indices were retained, as multilevel modelling is robust to missingness in estimation of model outcomes.

Models were tested in a step-wise approach, first including control variables (gender, age, level of education) and time. At the next step, physical exposure (PGA_{cum}) was added, followed by earthquake damage at time 1 and the increase of damage since time 1. The final model included the interaction between damage and time. Model fit

[6] As mentioned, we oversampled rural areas as well as the most heavily exposed areas. The geographical weighting was added to control for this overrepresentation.

For peer review only - http://bmjopen.bmj.com/site/about/guidelines.xhtml

was compared to assess which variables best predicted health outcomes. The best fitting models were those including the interaction of damage by time (see Table 3).

To highlight the implications of the findings, we distinguished poor and good health on the basis of health scores, enabling us to compute odds ratios (OR) and relative risk. For mental health we used the conventional criterion of MHI < 60 as cutoff[31]. For perceived health we classified “good” and “outstanding” as good health and all other scale points as poor (conform international convention). For symptoms we devised our own cutoff based on distributional characteristics combined with content criteria: A classification of < 60 as poor health resulted in 9% of the unaffected population being classified as such. Odds ratios were calculated in weighted models, controlling for age, education and gender.

Public involvement

The research setup (design and outcome measures) was discussed with an advisory board consisting of institutions (e.g., local municipalities) and representatives of the public (e.g., action groups). The present work has been disseminated in a public report.

RESULTS

Sample characteristics

There were no significant fluctuations in sample composition over time in terms of gender, education level and damage to own housing (see Table 2). Young respondents were underrepresented. There was attrition during the study. Dropout characteristics revealed no differences between exposed vs. control groups and no association between dropout and health. Analyses showed no indications that attrition

For peer review only - http://bmjopen.bmj.com/site/about/guidelines.xhtml

influenced any of the effects reported below. Over time, the average age of participants increased, as young people tended to have a higher likelihood of dropout. It is important to note that additional analyses found no significant interaction effect between age and exposure, suggesting that the effects of exposure were age-independent. Because the sample was not entirely representative and attrition relatively high, we carefully checked the potential consequences thereof and found no indications this influenced results.

Regarding levels of exposure, we know, based on existent data about damage per postal code[7], that the rates of exposure vary substantially within the region: in central areas up to 100% of homes have reported damage at least once. Outside these areas, there is progressively less damage. A substantial part of the province has (nearly) no damage. Average levels of damage are closely associated with ground motion[25]: In postal-code areas where 0% damage was reported until January 2016, there was hardly any exposure to ground motion (total ground motion PGA_{cum}=.07 mm/s^2). Only 3% of the sample located in this area suspected having damage due to earthquakes. In the areas where up to 20% damage was previously reported, ground motion levels were somewhat higher PGA_{cum}=0.64 mm/s$^{2)}$) and more people, 26% of the sample, indicated suspecting they have damage. And in the areas where 20% to 100% had reported damage, ground motion was considerably higher, PGA_{cum}=4.13 mm/s^2 and a very high percentage of our sample, 83%, suspected having damage.

The impact of exposure to gas extraction on health over time

[7] Provided by the institution handling damage claims, the Centrum voor Veilig Wonen

For peer review only - http://bmjopen.bmj.com/site/about/guidelines.xhtml

The analyses of conditional growth models on self-rated health, stress-related health problems and mental health showed consistent results across all three indicators. Table 3 shows the final results for all variables.

Important to note is that, after including control variables, there was a significant effect of exposure to physical ground motion (PGA_{cum}) on all three health indicators: more ground motion was associated with poorer health. The effect of time was also significant: over time, health deteriorated. In the next step, we included damage to housing. Importantly, the effect of ground motion was suppressed by the larger effects of exposure to multiple damage on all health indicators (p's<.01). This means that damage better predicts health outcomes than ground motion. Having damage once had no significant effect on any of the health indicators. Only participants with multiple damages experienced negative health consequences.

In step 3, the significant 'multiple damage (vs no damage) X time' interaction reveals that exposure to multiple damages is associated with a deterioration of health over time. The inclusion of this interaction variable improved model fit.

To interpret the effects and assess their magnitude, we calculated odds ratios for health measures at every time point, as well as the average impact of exposure over time (Table 4). Inhabitants exposed to damage once are only marginally (and not significantly) affected compared with a no-damage control group (averaged odds ratios range from 1.10 to 1.20). Those exposed to damage multiple times are more likely to report poor self-rated health (OR = 1.64, with a 95% confidence interval of 1.31;2.04), more stress-related health symptoms (OR = 2.52 [1.89;3.38]) and less good mental

For peer review only - http://bmjopen.bmj.com/site/about/guidelines.xhtml

health (OR = 1.83 [1.40;2.39]) than those without damage. This indicates that damage has a considerable impact on participants' health[8].

The table also suggests that differences between groups increase over time. Odds ratios for the difference between those with multiple damage and no damage are considerably higher 21 months after first measurement for self-rated health (OR = 2.00 [1.57;2.55]), mental health (OR = 2.38 [1.78;3.21]) and stress related health symptoms (OR = 3.36 [2.45;4.68]). In terms of relative risk, this means that those whose homes have multiple damage at T5 are 1.60[1.37;1.86] times more likely to report poor health, 2.11 [1.63;2.74] times more likely to report negative mental health and 2.84 [2.14;3.76] times more at risk of elevated levels of stress related health symptoms.

We also compared the weighted means of the ORs of control variables that are known correlates of health (age, gender, level of education) to the effect of damage (Figure 1). Across the three health measures, effect sizes (ORs) of damage are slightly larger than those of education (for a high versus low level of education, the average odds ratios over time are .53 [.41;.68] for self-rated health; OR = .58 [.42;.81] for mental health; and OR = .56 [.41;.79] for stress-related health symptoms).

DISCUSSION

Natural and induced seismicity can have negative consequences for local populations due to (acute or accumulated) health threats and irreversible changes to the living environment. Yet, so far studies have not assessed the accumulated impact of (the consequences of) induced seismicity on (psychosomatic) health *over time*.

---

[8] We also investigated whether women's health is affected differently by this stressor than men's, but as evidenced in Table S2 in the supplementary materials, this is not the case.

For peer review only - http://bmjopen.bmj.com/site/about/guidelines.xhtml

For peer review only

Moreover, most studies lack a non-exposure control group. The present study aimed to address these shortcomings by studying the impact of exposure to gas extraction (and subsequent damage to housing), compared to a no-exposure control group, on health over a time period of 21 months. Our study provides strong indications that exposure to negative side-effects of induced seismicity (e.g., damage to people's homes) constitutes an increasing health risk over time: We found that those exposed to multiple damage to housing experienced more negative health consequences than those without damage. Moreover, these effects increased over time. Results for exposure to ground motion were comparable.

To our knowledge, this is the only study of the long-term impact of induced seismicity on health. Therefore, we can only compare our results with the long-term impacts of very different types of disaster - limiting comparability. For one, the Chernobyl nuclear disaster: Study participants lived in a seriously contaminated area approximately 50 miles from Chernobyl. 6.5 years post disaster, inhabitants were twice as likely to have negative self-rated health (OR:2.25[1.96-2.58]) and psychological distress (OR:1.93[1.69-2.22]), compared to a non-exposed control group[32]. Chernobyl clearly constitutes a very different type of disaster and health risk (radiation exposure). The Brisbane floods were also very different in many respects (e.g., sudden disaster onset; deaths) but with some comparable outcomes, such as considerable damage to homes. 6-7 months post-disaster, those exposed to flooding were twice as likely to report psychological distress compared to the non-exposed[33]. It appears that the health impact of these very different and in many ways more 'acute' disasters are, in terms of effect size, somewhat comparable to the health impact of the more chronic

For peer review only - http://bmjopen.bmj.com/site/about/guidelines.xhtml
For peer review only

exposure to lower-level seismicity caused by gas extraction. One potential reason for the comparable effect sizes, is that our study focused not just on the environmental effect (e.g., amount of damage in a particular area) but zoomed in on the subgroup who were severely affected because they had multiple instances of damage to their own home. We further speculate that the man-made nature of the hazard (the fact that earthquakes are induced) may also enhance the impact on the population.

The present work also provides first time insights into the development of (psychosomatic) health symptoms in response to chronic disaster. In the area of acute disaster response, studies on longitudinal health impact reveal that distress decreases over time[34, 35], implying recovery of victims. Yet looking at discussions comparing chronic man-made (technological) disasters to acute natural disasters, we see reason to expect long term health impacts of the gas extraction: It contains elements that have been suggested as reasons for potential long-term health impact of technological disasters: A strong element of culpability in causing disaster, concerns about damage compensation after disaster and uncertainty regarding when disaster impact will end ("the book is never closed"; p.148, [12]; [36-38]. In line with this work, our findings suggest that for chronic disasters/hazards, negative effects can accumulate over time, presumably because the recurrent threat leads to an accumulation of stress.

Limitations

A potential limitation of this sample could be concerns about its representativeness: For one, Attrition was 45.3% over time and younger respondents were somewhat underrepresented. However, attrition was no different for the exposed and non-exposed groups, unrelated to health outcomes, and all further analyses

For peer review only - http://bmjopen.bmj.com/site/about/guidelines.xhtml

suggest that neither attrition nor sample characteristics had any substantial influence on results and conclusions drawn. Secondly, there might be an influence of confounding variables. Yet we believe effect sizes are robust: 1. The exposed and control groups were very similar regarding key population and geographical characteristics 2. Follow-up analyses revealed no interactions between any of the population characteristics and the effects of exposure.

Thirdly, responses could have been biased because participants knew the survey was about the social impact of gas extraction. It is relevant here that an 'objective' geographical exposure measure (peak ground acceleration) revealed comparable health outcomes to self-reported exposure. Moreover, analyses on a cross-sectional representative sample of residents (N=16340) in the 2016 health monitor of Statistics Netherlands, the National Institute for Public Health and public health services found comparable results. In this survey, the study intent was not clear.

One of the three health measures included, stress-related health symptoms, was an adaptation of a previously validated symptoms list[39], shortened for this specific study. Although the shortened version was not previously validated, it was psychometrically sound. Also, patterns are comparable across health measures, two of which are validated.

One of our exposure measures is self-reported damage. It is possible that damage is perceived differently depending on people's health status. Importantly, physical exposure to ground motion was associated with significant health effects. But effects of damage were stronger. This could be because damage is a more precise and proximate indicator of how individuals are affected by exposure, but also because of

For peer review only

recursive effects of (mental) health on perceived damage.

An important issue is generalizability: Is the situation in Groningen comparable to other areas with induced seismicity (e.g., fracking, wastewater injections)? We can only make reasoned inferences. Induced earthquakes are relatively common in energy projects which involve injection[40]. A priori, similar health consequences could occur in all sites in which populations are affected by induced earthquakes. Moreover, the vulnerability of people exposed to seismicity is likely influenced by similar factors: negative consequences are man-made and involve safety, health and social risks [12, 13]. In sum, although more research on the impact of induced seismicity is needed [41], we suggest effects are likely to generalize beyond the Groningen case.

Practical implications

The consequences of induced seismicity pose challenges to decision-makers. Benefits to the public good need to be balanced against the welfare of local populations [21]. As projects involving induced seismicity rapidly grow, governments and businesses face decisions whether to invest. Our work provides a case study of what occurs if seismicity is not kept in check. It can increase awareness of the vulnerability of exposed populations and provide important input for future decision making, monitoring and contingency planning.

Conclusion

Recent years have seen a rise in induced seismicity. Little is known about the (longitudinal) impact thereof on (psychosomatic) health. The present study is the first to our knowledge evidencing the long-term impact of induced seismicity on health.

For peer review only - http://bmjopen.bmj.com/site/about/guidelines.xhtml

Funding: This study was funded by the National Coordinator Groningen. Award/grant number: not applicable.

Contributorship: KS, BK, JR, JB, FO, FG and TP contributed to the research questions and study design. JR and FO contributed to data collection. BK and TP conducted statistical analyses. KS, BK and TP interpreted the results and wrote the initial draft of the manuscript. All authors commented on the final draft of the manuscript. BK and TP had full access to the data in the study and can take responsibility for the integrity of the data and the accuracy of the data analysis.

Conflict of Interest: The authors declare that they have no conflict of interest.

Ethical approval: All procedures performed in studies involving human participants were in accordance with the ethical standards of the ethical board of the department of psychology of the University of Groningen, The Netherlands (research code ppo-015-085).

Informed consent: Informed consent was obtained from all individual participants included in the study.

Data sharing statement: Data collected for this study are not available in order to guarantee anonimity of participants.

For peer review only

Table 1.
List of definitions

Conventional gas extraction	Extraction through drilling in deep subsoil reservoirs without the injection of chemical liquids.
Fracking	A well stimulation technique in which a rock is fractured by a pressured liquid.
Induced seismicity	Seismic events that are a result of human activity.
Natural seismicity	Seismic events that have a natural cause (e.g., volcanic eruption).
Peak ground acceleration	Measure of the largest increase in ground motion, recorded by a particular station during an earthquake.
Psychosomatic health	Health outcomes involving both mind and body.
Richter scale	Measure of strength of earthquakes with a logarithmic scale.
Shale gas	A natural gas that is trapped in fine grained sediment in rock.
Unconventional gas extraction	Gas reservoirs that require a special stimulation technique to extract gas (e.g., by injecting large quantities of partly chemical fluids underground).

For peer review only

Table 2

Demographic characteristics of participants participating in separate measurements: total number of participants participating in that measurement, decline of number of participants participating as compared to the number of participants participating at T1, mean age, distribution of level of education, distribution of personal exposure to damage due to gas extraction, distribution of gender, and amount of participants that completed the three health measures in that measurement. Netherlands 2016-2017

		T1 Feb '16	T2 June '16	T3 Nov '16	T4 Apr '17	T5 Nov '17
Total N		3934	3153	2638	2351	2150
Attrition (compared to T1)		-	19.9%	32.9%	40.2%	45.3%
Age (mean)		56.54	57.74	57.72	58.90	59.98
Level of education (N)	Low	968 (24.6%)	772 (24.5%)	616 (23.4%)	589 (25.1%)	535 (24.9%)
	Middle	1252 (31.8%)	970 (30.8%)	815 (30.9%)	713 (30.3%)	639 (29.7%)
	High	1533 (39.0%)	1238 (39.3%)	1068 (40.5%)	944 (40.2%)	852 (39.6%)
Gender (N)	Male	1967 (50.0%)	1547 (49.1%)	1306 (49.5%)	1182 (50.3%)	1068 (49.7%)
	Female	1849 (47.0%)	1480 (46.9%)	1231 (46.7%)	1097 (46.7%)	990 (46.0%)
Exposure to damage (N)	None	1477 (37.5%)	1204 (38.2%)	1027 (38.9%)	910 (38.7%)	846 (39.3%)
	One time	913 (23.2%)	626 (19.9%)	554 (21.0%)	505 (21.5%)	459 (21.3%)
	Multiple	1057 (26.9%)	1055 (33.5%)	940 (35.6%)	775 (33.0%)	736 (34.2%)
Perceived health (N)		3821 (97.1%)	-	2540 (96.3%)	2206 (93.8%)	2059 (95.8%)
Stress related health symptoms (N)		3767 (95.8%)	-	2533 (96.0%)	2206 (93.8%)	2045 (95.1%)
Mental health (N)		3711 (94.3%)	2819 (89.4%)	2501 (94.8%)	2179 (92.7%)	2021 (94.0%)

For peer review only - http://bmjopen.bmj.com/site/about/guidelines.xhtml

Table 3
Results of multilevel conditional growth models: *Unstandardized parameter estimates and standard errors for the association between time, damage, and the interaction between time and damage on perceived health, stress-related health symptoms, and mental health – adjusted for gender, age, level of education and ground motion (cumulative PGA). Netherlands 2016-2017.*

	Perceived health	Stress-related health symptoms[1]	Mental health
Gender	-0.05*	-5.40***	-2.68***
	(0.02)	(0.49)	(0.46)
Age	-0.01***	-0.02	0.07***
	(0.001)	(0.02)	(0.02)
Level of education (middle)	0.08*	0.61	1.01
	(0.03)	(0.67)	(0.62)
Level of education (high)	0.24***	3.02***	2.94***
	(0.03)	(0.63)	(0.59)
Cumulative PGA	-0.001	0.03	-0.01
	(0.004)	(0.09)	(0.08)
Time	-0.01	-0.25*	-0.49***
	(0.01)	(0.13)	(0.15)
Damage (one time)	-0.01	-0.46	-0.27
	(0.03)	(0.75)	(0.63)
Damage (multiple)	-0.12***	-4.31***	-3.35***
	(0.03)	(0.76)	(0.65)
Time * Damage (one time)	-0.02	-0.13	-0.07
	(0.01)	(0.20)	(0.24)
Time * Damage (multiple)	-0.03***	-0.45*	-0.60**
	(0.01)	(0.19)	(0.23)
Constant	3.86***	80.19***	77.78***
	(0.03)	(0.67)	(0.60)
Observations	10,256	9,100	9,686
Log Likelihood	-10,104.58	-36,205.01	-38,020.51
Akaike Inf. Crit.	20,239.16	72,440.02	76,071.02
Bayesian Inf. Crit.	20,347.69	72,546.76	76,178.69

Note. *p<0.05; **p<0.01; ***p<0.001
1. Stress-related health symptoms were reverse-coded such that higher levels indicate less stress

For peer review only - http://bmjopen.bmj.com/site/about/guidelines.xhtml

Table 4

Proportion of participants who have poor health and OR of participants who have poor health with damage (compared to those with no damage) across measurements, with 95% confidence intervals – adjusted for age, gender and level of education. Netherlands 2016-2017

Measurement	Damage	Percentage poor health	Odds ratio
Self-rated health			
T1	None	22.2% [19.9%;24.5%]	-
	One time	22.5% [19.6%;25.4%]	1.02 [0.82;1.26]
	Multiple	25.6% [22.7%;28.4%]	1.21 [0.99;1.47]
T3	None	21.6% [18.8%;24.3%]	-
	One time	24.4% [20.5%;28.3%]	1.17 [0.90;1.53]
	Multiple	32.4% [29.2%;35.7%]	1.75 [1.41;2.18]
T4	None	21.3% [18.4%;24.2%]	-
	One time	30.0% [25.7%;34.4%]	1.60 [1.22;2.09]
	Multiple	35.5% [31.8%;39.2%]	2.06 [1.63;2.61]
T5	None	23.6% [20.3%;26.9%]	-
	One time	27.5% [22.9%;32.1%]	1.23 [0.92;1.65]
	Multiple	38.0% [34.0%;42.0%]	2.00 [1.57;2.55]
Weighted average	None	22.1% [19.4%;24.9%]	-
	One time	25.6% [21.8%;29.4%]	1.20 [0.93;1.55]
	Multiple	31.8% [28.5%;35.2%]	1.64 [1.31;2.04]
Symptoms			
T1	None	9.2% [7.7%;10.8%]	-
	One time	10.0% [7.9%;12.1%]	1.09 [0.81;1.47]
	Multiple	17.3% [14.9%;19.7%]	2.08 [1.62;2.68]
T3	None	7.1% [5.5%;8.8%]	-
	One time	6.5% [4.3%;8.6%]	0.90 [0.58;1.37]
	Multiple	13.7% [11.4%;16.1%]	2.09 [1.55;2.85]
T4	None	8.1% [6.2%;10.0%]	-
	One time	9.4% [6.7%;12.1%]	1.18 [0.78;1.75]
	Multiple	21.8% [18.7%;25.0%]	3.24 [2.40;4.42]
T5	None	7.1% [5.3%;9.0%]	-
	One time	9.1% [6.3%;11.9%]	1.30 [0.84;1.99]
	Multiple	20.3% [17.0%;23.5%]	3.36 [2.45;4.68]
Weighted average	None	8.0% [6.4%;9.8%]	-
	One time	8.8% [6.5%;11.2%]	1.10 [0.75;1.60]
	Multiple	18.0% [15.3%;20.7%]	2.52 [1.89;3.38]
Mental health			

For peer review only

T1	None	8.5% [7.0%;10.0%]	-
	One time	9.0% [7.0%;10.9%]	1.06 [0.78;1.43]
	Multiple	12.4% [10.3%;14.5%]	1.53 [1.18;1.98]
T2	None	8.5% [6.8%;10.2%]	-
	One time	9.2% [6.9%;11.6%]	1.09 [0.77;1.54]
	Multiple	18.1% [15.6%;20.7%]	2.40 [1.86;3.13]
T3	None	11.1% [9.0%;13.2%]	-
	One time	12.0% [9.2%;14.9%]	1.10 [0.79;1.51]
	Multiple	14.5% [12.1%;16.9%]	1.36 [1.04;1.77]
T4	None	11.9% [9.6%;14.1%]	-
	One time	11.8% [8.9%;14.7%]	0.99 [0.71;1.38]
	Multiple	20.3% [17.2%;23.4%]	1.9 [1.46;2.47]
T5	None	9.0% [6.9%;11.1%]	-
	One time	12.5% [9.3%;15.7%]	1.44 [0.99;2.07]
	Multiple	19.1% [15.9%;22.2%]	2.38 [1.78;3.21]
Weighted average	None	9.7% [7.8%;11.5%]	-
	One time	10.6% [8.1%;13.2%]	1.11 [0.80;1.55]
	Multiple	16.4% [13.8%;19.0%]	1.83 [1.40;2.39]

Note. Scores were categorised as low health as follows: 1) very poor, poor, or fair perceived health; 2) a score below 60 for stress related health symptoms, and 3) a score below 60 for mental health.

For peer review only

For peer review only - http://bmjopen.bmj.com/site/about/guidelines.xhtml

REFERENCES

1 Konkel L. In the Neighborhood of 18 Million: Estimating How Many People Live Near Oil and Gas Wells, *Environ Health Perspect* 2017;125:UNSP 124003 doi:10.1289/EHP2553 [published Online First: DEC].

2 U.S. Energy Information Administration. United States Shale gas reserves per state. 2017;2019.

3 Dai W, Chen L, Lai Z, et al. The incidence of post-traumatic stress disorder among survivors after earthquakes: A systematic review and meta-analysis, *BMC Psychiatry* 2016;16.

4 Neria Y, Nandi A, Galea S. Post-traumatic stress disorder following disasters: A systematic review, *Psychol Med* 2008;38:467-80 doi:10.1017/S0033291707001353.

5 Beaglehole B, Mulder RT, Frampton CM, et al. Psychological distress and psychiatric disorder after natural disasters: systematic review and meta-analysis, *The British Journal of Psychiatry* 2018;213:716-22.

6 Goldmann E, Galea S. Mental health consequences of disasters, *Annu Rev Public Health* 2014;35:169-83.

7 Galea S, Nandi A, Vlahov D. The epidemiology of post-traumatic stress disorder after disasters, *Epidemiol Rev* 2005;27:78-91.

8 Norris FH, Friedman MJ, Watson PJ. 60,000 disaster victims speak: Part II. Summary and implications of the disaster mental health research, *Psychiatry: Interpersonal and biological processes* 2002;65:240-60.

9 Rubonis AV, Bickman L. Psychological impairment in the wake of disaster: The disaster–psychopathology relationship. *Psychol Bull* 1991;109:384.

10 Keranen KM, Weingarten M. Induced Seismicity, *Annual Review of Earth and Planetary Sciences, Vol 46* 2018;46:149-74 doi:10.1146/annurev-earth-082517-010054.

11 van der Elst, Nicholas J., Page MT, Weiser DA, et al. Induced earthquake magnitudes are as large as (statistically) expected, *Journal of Geophysical Research-Solid Earth* 2016;121:4575-90 doi:10.1002/2016JB012818 [published

For peer review only

Online First: JUN].
12 Couch SR, Kroll-Smith JS. The chronic technical disaster: Toward a social scientific perspective, *Social Science Quarterly* 1985;66:564.
13 Sangaramoorthy T, Jamison AM, Boyle MD, et al. Place-based perceptions of the impacts of fracking along the Marcellus Shale, *Soc Sci Med* 2016;151:27-37.
14 Rabinowitz PM, Slizovskiy IB, Lamers V, et al. Proximity to Natural Gas Wells and Reported Health Status: Results of a Household Survey in Washington County, Pennsylvania, *Environ Health Perspect* 2015;123:21-6 doi:10.1289/ehp.1307732.
15 Steinzor N, Subra W, Sumi L. Investigating links between shale gas development and health impacts through a community survey project in Pennsylvania. *New solutions : a journal of environmental and occupational health policy : NS* 2013;23:55-83 doi:10.2190/NS.23.1.e.
16 Tustin AW, Hirsch AG, Rasmussen SG, et al. Associations between Unconventional Natural Gas Development and Nasal and Sinus, Migraine Headache, and Fatigue Symptoms in Pennsylvania, *Environ Health Perspect* 2017;125:189-97 doi:10.1289/EHP281.
17 Weinberger B, Greiner LH, Walleigh L, et al. Health symptoms in residents living near shale gas activity: A retrospective record review from the Environmental Health Project. *Preventive medicine reports* 2017;8:112-5 doi:10.1016/j.pmedr.2017.09.002.
18 Wilson MP, Foulger GR, Gluyas JG, et al. HiQuake: The human-induced earthquake database, *Seismol Res Lett* 2017;88:1560-5.
19 Aczel MR, Makuch KE. Shale, Quakes, and High Stakes: Regulating Fracking-Induced Seismicity in Oklahoma, USA and Lancashire, UK, *Case Studies in the Environment* 2019.
20 Keranen KM, Weingarten M, Abers GA, et al. Sharp increase in central Oklahoma seismicity since 2008 induced by massive wastewater injection, *Science* 2014;345:448-51 doi:10.1126/science.1255802 [published Online First: JUL 25].
21 Fleming RC, Reins L. Shale gas extraction, precaution and prevention: A conversation on regulatory responses, *Energy Research & Social Science* 2016;20:131-41 doi:10.1016/j.erss.2016.05.013 [published Online First: OCT].

For peer review only

For peer review only - http://bmjopen.bmj.com/site/about/guidelines.xhtml

22 Vlek C. Rise and reduction of induced earthquakes in the Groningen gas field, 1991-2018: statistical trends, social impacts, and policy change, *Environ Earth Sci* 2019;78:59 doi:10.1007/s12665-019-8051-4 [published Online First: FEB].

23 Dutch Safety Board. Earthquake risks in Groningen. Research concerning the role population safety played in decision making procedures about gas extraction (1959-2014) , 2015.

24 Postmes T, Stroebe K, Richardson J, et al. Consequences of ground motion for the Groningen population: Experienced safety, health and future perspective 2016-2017. *University of Groningen* 2018.

25 Geurts C, Steenbergen R. Note for the Dutch State Supervision of Mines: The relation between Peak Ground Acceleration and the risk of damage for induced earthquakes in Groningen. *The Netherlands Organisation for Applied Scientific Research* 2016.

26 Pappas G. Health interview surveys: Towards international harmonization of methods and instruments - deBruin,A, Picavet,HSJ, Nossikoy,A, *Soc Sci Med* 1997;44:1431-2 doi:10.1016/S0277-9536(97)84078-6 [published Online First: MAY].

27 Ware JE, Gandek B, IQOLA Project. Overview of the SF-36 Health Survey and the International Quality of Life Assessment (IQOLA) Project, *J Clin Epidemiol* 1998;51:903-12 doi:10.1016/S0895-4356(98)00081-X [published Online First: NOV].

28 Van den Berg B, Yzermans CJ, Van der Velden, Peter G., et al. Risk Factors for Unexplained Symptoms After a Disaster: A Five-Year Longitudinal Study in General Practice, *Psychosomatics* 2009;50:69-77 doi:10.1176/appi.psy.50.1.69.

29 Berwick DM, Murphy JM, Goldman PA, et al. Performance of a five-item mental health screening test. *Med Care* 1991.

30 Raudenbush SW, Bryk AS. Hierarchical linear models: Applications and data analysis methods: Sage 2002.

31 Rumpf H, Meyer C, Hapke U, et al. Screening for mental health: validity of the MHI-5 using DSM-IV Axis I psychiatric disorders as gold standard, *Psychiatry Res* 2001;105:243-53.

For peer review only - http://bmjopen.bmj.com/site/about/guidelines.xhtml

32 Havenaar J, Rumyantzeva G, Kasyanenko A, et al. Health effects of the Chernobyl disaster: Illness or illness behavior? A comparative general health survey in two former Soviet regions, *Environ Health Perspect* 1997;105:1533-7 doi:10.2307/3433666 [published Online First: DEC].

33 Alderman K, Turner LR, Tong S. Assessment of the Health Impacts of the 2011 Summer Floods in Brisbane, *Dis Med Public Health Prep* 2013;7:380-6 doi:10.1017/dmp.2013.42 [published Online First: AUG].

34 Lowell A, Suarez-Jimenez B, Helpman L, et al. 9/11-related PTSD among highly exposed populations: a systematic review 15 years after the attack, *Psychol Med* 2018;48:537-53 doi:10.1017/S0033291717002033 [published Online First: MAR].

35 van der Velden, Peter G., Wong A, Boshuizen HC, et al. Persistent mental health disturbances during the 10years after a disaster: Four-wave longitudinal comparative study, *Psychiatry Clin Neurosci* 2013;67:110-8 doi:10.1111/pcn.12022 [published Online First: FEB].

36 Baum A. Implications of psychological research on stress and technological accidents. *Am Psychol* 1993;48:665.

37 Picou JS. The “talking circle” as sociological practice: Cultural transformation of chronic disaster impacts, *Sociological Practice* 2000;2:77-97.

38 Picou JS, Marshall BK, Gill DA. Disaster, litigation, and the corrosive community, *Social forces* 2004;82:1493-522.

39 van den Berg B, Grievink L, Yzermans J, et al. Medically unexplained physical symptoms in the aftermath of disasters, *Epidemiol Rev* 2005;27:92-106 doi:10.1093/epirev/mxi001.

40 Ellsworth WL. Injection-induced earthquakes, *Science* 2013;341:1225942.

41 Hirsch JK, Smalley KB, Selby-Nelson EM, et al. Psychosocial Impact of Fracking: a Review of the Literature on the Mental Health Consequences of Hydraulic Fracturing, *International Journal of Mental Health and Addiction* 2018;16:1-15 doi:10.1007/s11469-017-9792-5 [published Online First: FEB].

For peer review only

Figure 1. Weighted average odds ratios.

For peer review only

For peer review only - http://bmjopen.bmj.com/site/about/guidelines.xhtml

Figure 1. Weighted average odds ratios.

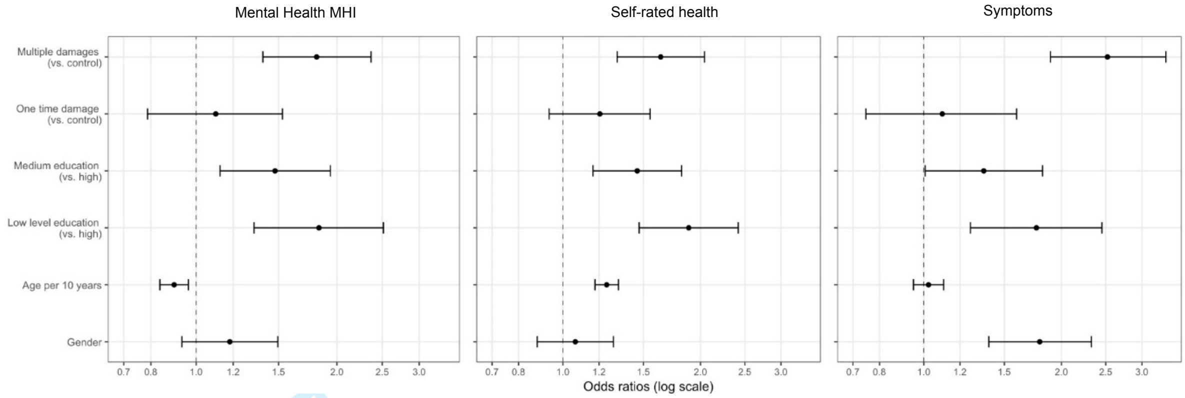

For peer review only

Supplementary materials

Seismicity in the Groningen gasfield

Figure S1: Number of earthquakes and volume of gas production in the Groningen gasfield:

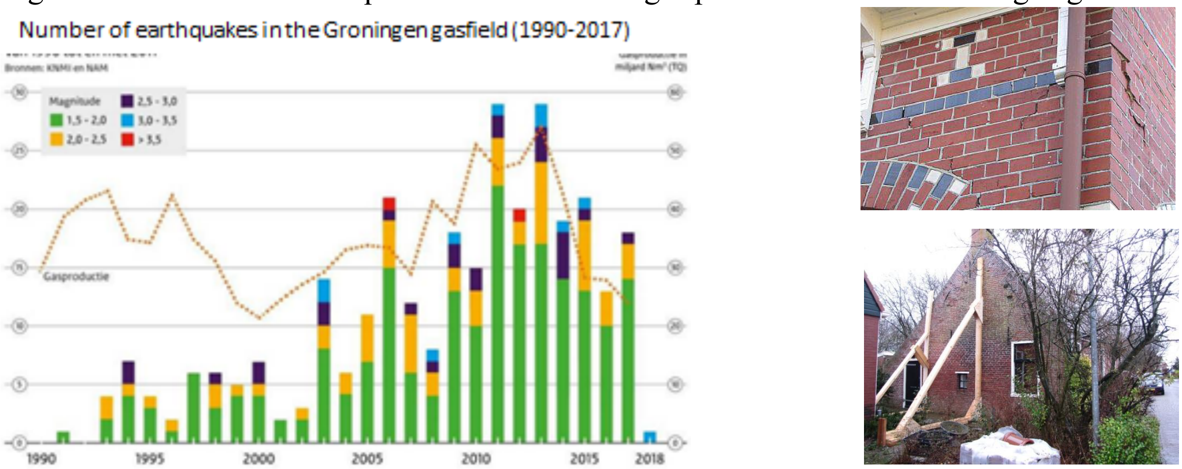

The volume of gas production in the subsoil of Groningen is presented as a dotted line in Figure 2. The number of earthquakes magnitude 2 or higher increased from 2003 onwards. The earthquake of August 2012 in the village of Huizinge (magnitude 3,6 on Richter scale) was a landmark event that caused unrest and great concern among the population in the province of Groningen. It was the heaviest earthquake measured in the province. The magnitude of earthquakes is recorded by the Dutch national borehole network, the regional accelerometer network and all additional seismic stations in the south of the Netherlands. European seismic stations reported the event at epicentral distances up to 800 km (Dost and Kraaipoel, 2012).

410,000 residents of the province of Groningen are exposed to these induced earthquakes: they live in a postcode area where damage has been recognized by the oil company responsible (NAM, a joint venture of Shell and Exxon). Of these, 134,363 adults report having damage to their property (Postmes et al., 2017). Of these 68,343 report having damage multiple times.

Looking worldwide, there are 1174 locations in which induced seismicity is taking place. 11% of seismicity is due to conventional oil or gas extraction (see inducedearthquakes.org/)

References

Dost B and Kraaipoel D. The August 16, 2012 earthquake near Huizinge in Groningen province. Royal Dutch Meteorological Institute, 2013.

Postmes, T., Stroebe, K., Richardson, J., LeKander, B., Oldersma, F., Broer, J. & Greven, F. (2018). Gevolgen van bodembeweging voor Groningers: Ervaren veiligheid, gezondheid en toekomstperspectief 2016-2017. Groningen: Heymans Instituut, Rijksuniversiteit Groningen.

review only

Table S1

Demographic characteristics of participants completing separate measurements per level of damage: total sample size, mean age, distribution of level of education, distribution of level of damage, distribution of gender, and amount of participants that completed the health measures. Netherlands 2016-2017

Damage to house at T1			T1 Feb '16	T2 June '16	T3 Nov '16	T4 Apr '17	T5 Nov '17
None	Total N		1477	1166	968	886	801
	Age (mean)		57.67	59.13	59.13	60.28	61.48
	Level of education (N)	Low	430	340	266	267	238
		Middle	460	351	290	255	226
		High	562	453	396	349	324
	Gender (N)	Male	794	621	515	471	427
		Female	683	545	453	415	374
	Perceived health (N)		1467	-	934	835	784
	Stress related health symptoms (N)		1452	-	937	836	780
	Mental health (N)		1432	1048	920	828	769
One time	Total N		913	730	608	559	490
	Age (mean)		58.32	58.87	58.81	60.06	60.86
	Level of education (N)	Low	237	191	155	143	131
		Middle	295	235	195	182	159
		High	363	295	250	227	193
	Gender (N)	Male	505	398	345	323	279
		Female	407	332	263	236	211
	Perceived health (N)		907	-	587	521	464
	Stress related health symptoms (N)		894	-	584	522	463
	Mental health (N)		895	666	581	517	456
Multiple	Total N		1057	825	704	609	558
	Age (M)		54.06	55.57	55.60	56.71	57.70
	Level of education (N)	Low	215	168	133	120	110
		Middle	381	289	246	213	188
		High	445	356	315	268	253
	Gender (N)	Male	493	385	323	284	269
		Female	563	440	381	325	289
	Perceived health (N)		1048	-	683	578	537
	Stress related health symptoms (N)		1041	-	675	577	530
	Mental health (N)		1018	739	674	570	528

For peer review only

Table S2

Unstandardized regression parameter estimates and standard errors for the association between time, damage, and the interaction between time and damage on perceived health, stress-related health symptoms, and mental health, and the interaction between gender and damage on perceived health, stress-related health symptoms, and mental health – adjusted for gender, age, level of education and ground motion (cumulative PGA). Netherlands 2016-2017.

	Perceived health	Stress-related health symptoms[1]	Mental health
Gender	-0.07	-5.08***	-2.81***
	(0.04)	(0.75)	(0.70)
Age	-0.01***	-0.02	0.07***
	(0.001)	(0.02)	(0.02)
Level of education (middle)	0.08*	0.62	0.99
	(0.03)	(0.67)	(0.62)
Level of education (high)	0.23***	3.03***	2.92***
	(0.03)	(0.63)	(0.59)
Cumulative PGA	-0.001	0.03	-0.01
	(0.004)	(0.09)	(0.08)
Time	-0.01	-0.25*	-0.49***
	(0.01)	(0.13)	(0.15)
Damage (one time)	-0.02	-0.19	-0.05
	(0.04)	(0.93)	(0.81)
Damage (multiple)	-0.14**	-4.04***	-3.79***
	(0.04)	(0.95)	(0.84)
Time * Damage (one time)	-0.02	-0.13	-0.07
	(0.01)	(0.20)	(0.24)
Time * Damage (multiple)	-0.03***	-0.45*	-0.60**
	(0.01)	(0.19)	(0.23)
Gender * Damage (one time)	0.03	-0.58	-0.51
	(0.06)	(1.21)	(1.13)
Gender * Damage (multiple)	0.04	-0.55	0.85
	(0.05)	(1.15)	(1.07)
Constant	3.87***	80.04***	77.86***
	(0.03)	(0.73)	(0.65)
Observations	10,256	9,100	9,686
Log Likelihood	-10,104.34	-36,204.85	-38,019.84
Akaike Inf. Crit.	20,242.68	72,443.70	76,073.68
Bayesian Inf. Crit.	20,365.69	72,564.67	76,195.71

Note. *p<0.05; **p<0.01; ***p<0.001

1. Stress-related health symptoms were reverse-coded such that higher levels indicate less stress

STROBE Statement—checklist of items that should be included in reports of observational studies

	Item No.	Recommendation	Page No.	Relevant text from manuscript
**Title and abstract**	1	(*a*) Indicate the study's design with a commonly used term in the title or the abstract	1/2	Title is mentioned on page 1
		(*b*) Provide in the abstract an informative and balanced summary of what was done and what was found	2	
**Introduction**				
Background/rationale	2	Explain the scientific background and rationale for the investigation being reported	4/5	
Objectives	3	State specific objectives, including any prespecified hypotheses	5/6	The present work was designed to address the lack of information regarding the long-term impact of induced seismicity for residents: It studies the longitudinal (psychosomatic) health impact of induced seismicity on a group exposed to the consequences of seismicity (damage to housing) versus a control group not exposed to these consequences." "The present study is novel in charting the chronic impact of exposure to damage on health over a time period of almost two years, on a large sample. We tested the following hypotheses: 1. Exposure versus

For peer review only - http://bmjopen.bmj.com/site/about/guidelines.xhtml

				non-exposure will have a negative impact on (psychosomatic) health outcomes. 2. Increases in exposure are related to poorer health outcomes.”
**Methods**				
Study design	4	Present key elements of study design early in the paper	5-8	
Setting	5	Describe the setting, locations, and relevant dates, including periods of recruitment, exposure, follow-up, and data collection	6-8	“A stratified random sample was drawn of 25000 residents of the province of Groningen, aged 16 and over, from the official municipal population records which is a complete register of all legal residents. Sampling occurred in areas where damage is reported and from outlying areas where this is not the case. Postal-code areas that were rural and strongly affected by damage were oversampled . Residents received letters with personal login codes and one reminder. Eighteen percent (N=4577) signed up for the study, and later received invitations to all questionnaires. Of these 4577, 86% (3934) filled out the first questionnaire.”; “Questionnaires were sent via

For peer review only

				an email link or by post. A reminder was sent after 2 weeks. Participants (T1: N=3934; T5: N=2150) completed measures at 5 time points during 2 years (T1: February 2016, T2: June 2016; T3: November 2016; T4: April 2017; T5: November 2017; see Table 2).
Participants	6	(*a*) *Cohort study*—Give the eligibility criteria, and the sources and methods of selection of participants. Describe methods of follow-up<br>*Case-control study*—Give the eligibility criteria, and the sources and methods of case ascertainment and control selection. Give the rationale for the choice of cases and controls<br>*Cross-sectional study*—Give the eligibility criteria, and the sources and methods of selection of participants	7	
		(*b*) *Cohort study*—For matched studies, give matching criteria and number of exposed and unexposed<br>*Case-control study*—For matched studies, give matching criteria and the number of controls per case		
Variables	7	Clearly define all outcomes, exposures, predictors, potential confounders, and effect modifiers. Give diagnostic criteria, if applicable	8-10	
Data sources/ measurement	8*	For each variable of interest, give sources of data and details of methods of assessment (measurement). Describe comparability of assessment methods if there is more than one group	8-10	
Bias	9	Describe any efforts to address potential sources of bias	10	Analyses controlled for age, gender and education level. Analyses were weighted to correct for sampling effects of age, gender and degree of exposure of postal-code areas . The weights were developed to

For peer review only

				counteract any potential distortive effect due to age composition, among others (e.g., because younger people were underrepresented, see results section). We report the weighted results. The unweighted results were very similar.
Study size	10	Explain how the study size was arrived at	7	

Continued on next page

For peer review only - http://bmjopen.bmj.com/site/about/guidelines.xhtml

Quantitative variables	11	Explain how quantitative variables were handled in the analyses. If applicable, describe which groupings were chosen and why	10-11	
Statistical methods	12	(*a*) Describe all statistical methods, including those used to control for confounding	10-11	
		(*b*) Describe any methods used to examine subgroups and interactions	10-11	
		(*c*) Explain how missing data were addressed	10	Participants with missing data on the health indices were retained, as multilevel modelling is robust to missingness in estimation of model outcomes. See Table 2 for an overview of the number of participants completing each health measure per time point.
		(*d*) *Cohort study*—If applicable, explain how loss to follow-up was addressed<br>*Case-control study*—If applicable, explain how matching of cases and controls was addressed<br>*Cross-sectional study*—If applicable, describe analytical methods taking account of sampling strategy	11-12	There was attrition during the study. Dropout characteristics revealed no differences between exposed vs. control groups and no association between dropout and health. Analyses showed no indications that attrition influenced any of the effects reported below. Over time, the average age of participants increased, as young people tended to have a higher likelihood of dropout. It is important to note that additional analyses found no significant interaction effect between age and exposure, suggesting that the effects of exposure were age-independent. Because the sample was not entirely representative and attrition relatively high, we carefully checked the potential consequences thereof and found no indications this influenced results.
		(*e*) Describe any sensitivity analyses		
**Results**				

For peer review only - http://bmjopen.bmj.com/site/about/guidelines.xhtml

Participants	13*	(a) Report numbers of individuals at each stage of study—eg numbers potentially eligible, examined for eligibility, confirmed eligible, included in the study, completing follow-up, and analysed		Table 2
		(b) Give reasons for non-participation at each stage	N/A	
		(c) Consider use of a flow diagram	N/A	
Descriptive data	14*	(a) Give characteristics of study participants (eg demographic, clinical, social) and information on exposures and potential confounders		Table 2
		(b) Indicate number of participants with missing data for each variable of interest	N/A	
		(c) *Cohort study*—Summarise follow-up time (eg, average and total amount)		
Outcome data	15*	*Cohort study*—Report numbers of outcome events or summary measures over time		
		Case-control study—Report numbers in each exposure category, or summary measures of exposure		
		Cross-sectional study—Report numbers of outcome events or summary measures		
Main results	16	(*a*) Give unadjusted estimates and, if applicable, confounder-adjusted estimates and their precision (eg, 95% confidence interval). Make clear which confounders were adjusted for and why they were included	13-14	Table 3/4
		(*b*) Report category boundaries when continuous variables were categorized	11	Tables 3/4
		(*c*) If relevant, consider translating estimates of relative risk into absolute risk for a meaningful time period		

Continued on next page

For peer review only

Other analyses	17	Report other analyses done—eg analyses of subgroups and interactions, and sensitivity analyses	
**Discussion**			
Key results	18	Summarise key results with reference to study objectives	15
Limitations	19	Discuss limitations of the study, taking into account sources of potential bias or imprecision. Discuss both direction and magnitude of any potential bias	16-17
Interpretation	20	Give a cautious overall interpretation of results considering objectives, limitations, multiplicity of analyses, results from similar studies, and other relevant evidence	16
Generalisability	21	Discuss the generalisability (external validity) of the study results	17-18
**Other information**			
Funding	22	Give the source of funding and the role of the funders for the present study and, if applicable, for the original study on which the present article is based	19

*Give information separately for cases and controls in case-control studies and, if applicable, for exposed and unexposed groups in cohort and cross-sectional studies.

Note: An Explanation and Elaboration article discusses each checklist item and gives methodological background and published examples of transparent reporting. The STROBE checklist is best used in conjunction with this article (freely available on the Web sites of PLoS Medicine at http://www.plosmedicine.org/, Annals of Internal Medicine at http://www.annals.org/, and Epidemiology at http://www.epidem.com/). Information on the STROBE Initiative is available at www.strobe-statement.org.

For peer review only

BMJ Open

Chronic disaster impact: the long-term psychological and physical health consequences of housing damage due to induced earthquakes

Journal:	*BMJ Open*
Manuscript ID	bmjopen-2020-040710.R2
Article Type:	Original research
Date Submitted by the Author:	07-Apr-2021
Complete List of Authors:	Stroebe, Katherine; Rijksuniversiteit Groningen, Social Psychology<br>Kanis, Babet; Rijksuniversiteit Groningen, Social Psychology<br>Richardson, Justin; Rijksuniversiteit Groningen, Social Psychology<br>Oldersma, Frans; Department for Statistics and Research, Municipality of Groningen, Groningen, Netherlands<br>Broer, Jan; Municipal Health Services Groningen, ABPG;<br>Greven, Frans; Municipal Health Services Groningen, the Netherlands, Department of Environmental Health<br>Postmes, Tom; Rijksuniversiteit Groningen, Social Psychology
**Primary Subject Heading**:	Public health
Secondary Subject Heading:	Mental health, Public health
Keywords:	MENTAL HEALTH, EPIDEMIOLOGY, PUBLIC HEALTH

SCHOLARONE™
Manuscripts

I, the Submitting Author has the right to grant and does grant on behalf of all authors of the Work (as defined in the below author licence), an exclusive licence and/or a non-exclusive licence for contributions from authors who are: i) UK Crown employees; ii) where BMJ has agreed a CC-BY licence shall apply, and/or iii) in accordance with the terms applicable for US Federal Government officers or employees acting as part of their official duties; on a worldwide, perpetual, irrevocable, royalty-free basis to BMJ Publishing Group Ltd ("BMJ") its licensees and where the relevant Journal is co-owned by BMJ to the co-owners of the Journal, to publish the Work in this journal and any other BMJ products and to exploit all rights, as set out in our licence.

The Submitting Author accepts and understands that any supply made under these terms is made by BMJ to the Submitting Author unless you are acting as an employee on behalf of your employer or a postgraduate student of an affiliated institution which is paying any applicable article publishing charge ("APC") for Open Access articles. Where the Submitting Author wishes to make the Work available on an Open Access basis (and intends to pay the relevant APC), the terms of reuse of such Open Access shall be governed by a Creative Commons licence – details of these licences and which Creative Commons licence will apply to this Work are set out in our licence referred to above.

Other than as permitted in any relevant BMJ Author's Self Archiving Policies, I confirm this Work has not been accepted for publication elsewhere, is not being considered for publication elsewhere and does not duplicate material already published. I confirm all authors consent to publication of this Work and authorise the granting of this licence.

For peer review only

Chronic disaster impact: the long-term psychological and physical health consequences of housing damage due to induced earthquakes

Stroebe, K.[1], Kanis, B.[1], Richardson, J.[1], Oldersma, F.[2], Broer, J.[3], Greven, F.[3] & Postmes, T.[1],

1. University of Groningen, Faculty of Behavioral and Social Sciences, Groningen, Netherlands
2. Department for Statistics and Research, Municipality of Groningen, Groningen, Netherlands
3. Municipal Health Services Groningen, Groningen, Netherlands

Corresponding author: Katherine Stroebe, k.e.stroebe@rug.nl, +313636509

For peer review only

Abstract

Objectives: To evaluate the long-term (psychosomatic) health consequences of man-made earthquakes compared to a non-exposure control group. Exposure was hypothesized to have an increasingly negative impact on health outcomes over time.

Setting — Large scale gas extraction in the Netherlands causing earthquakes and considerable damage.

Participants — A representative sample of inhabitants randomly selected from municipal population records; contacted 5 times during 21 months (T1:N=3934; T5:N=2150; mean age: 56.54; 50%males; At T5, N=846 (39.3%) had no, 459 (21.3%) once, and 736 (34.2%) repeated damages.

Main measures — (Psychosomatic) health outcomes: Self rated health and Mental Health Inventory (both: validated; Short Form Health Survey); stress related health symptoms (shortened version of previously validated symptoms list) Independent variable: Exposure to the consequences of earthquakes assessed via physical (Peak Ground Acceleration, PGA) and personal exposure (damage to housing: none, once, repeated).

Results: Exposure to induced earthquakes has negative health consequences especially for those whose homes were damaged repeatedly. Compared to a no-damage control group, repeated damage was associated with lower self-rated health (OR:1.64), mental health (OR:1.83) and more stress-related health symptoms (OR:2.52). Effects increased over time: In terms of relative risk, by T5, those whose homes had repeated damage were respectively 1.60 and 2.11 times more likely to report poor health and negative mental health and 2.84 times more at risk of elevated stress related health symptoms. Results for physical exposure were comparable.

Conclusion

This is the first study to provide evidence that induced earthquakes can have negative health consequences for inhabitants over time. It identifies the subpopulation particularly at risk: people with repeated damages who have experienced many earthquakes. Findings can have important implications for the prevention of negative health consequences of induced earthquakes.

For peer review only - http://bmjopen.bmj.com/site/about/guidelines.xhtml

Keywords: induced earthquakes; seismicity; longitudinal; psychosomatic health, gas extraction

Strengths and limitations

- The present study employs a longitudinal panel design with 5 measurement points to study (pschosomatic) health consequences of manmade earthquakes caused by gas extraction
- The study has an exposed (residents with damage to housing) and a non-exposed (residents with no damage) control group
- Two health measures (self-rated health; Mental Health Inventory) were previously validated, the third was an adaptation of a previously validated symptoms list
- Younger respondents were somewhat underrepresented in our sample
- There was 45.3% attrition over time but attrition was no different for the exposed versus non-exposed groups and was unrelated to health outcomes.

For peer review only

INTRODUCTION

Recent years have seen a rise in induced seismicity due to human activities such as fracking, mining or gas extraction. This development is expected to continue. While smaller in magnitude than natural seismicity, induced seismicity can expose populations to considerable physical (e.g., damage to housing) and social risks (e.g., conflicts between residents and institutions). Moreover, this exposure is recurrent and chronic over time. While there is some insight into the long-term health risks of naturally occurring seismicity, little is known about the impact of induced seismicity. Given the increased use of energy technologies associated with seismicity, also in densely populated areas, knowledge of its health impact is important [1, 2] (see also Table 1 for definitions of gas-extraction related terminology).

Naturally occurring seismicity is associated with mental health problems in survivors (e.g., depression, PTSD)[3-5]. While (some) more studies have been considering the longitudinal health effects of seismicity, lack of longitudinal design and an unexposed control group have been highlighted as major concerns for studies of natural disasters [3, 5, 6]. Moreover, the impact of natural- cannot be equated with that of induced- seismicity for several reasons: Systematic reviews suggest there is *lower* prevalence of mental health impairment for natural compared to human/technological disasters [7, 8]; but see [9]. Additionally, different stressors are at play: Natural seismicity can be of greater magnitude, causing death and extensive damages to buildings. For induced seismicity, the maximum magnitude of earthquakes tends to be smaller [10, 11]. Risks involve damage to property and an incremental impact on health,

For peer review only - http://bmjopen.bmj.com/site/about/guidelines.xhtml

as residents are exposed to long-term stressors (e.g., damages; changing community relations; conflicts of interest with powerful institutions [12, 13]).

Factual information regarding the health impact of induced seismicity is sparse. Cross-sectional self-report studies [14-16] and an evaluation of health records of exposed adults[17] in the context of unconventional gas extraction, suggest associations between induced seismicity and increased (psychosomatic) health symptoms (e.g., sleep disruption, headaches, stress). It is difficult to draw conclusions regarding the impact of seismicity from such studies: Exposure to (the consequences of) seismicity is not distinguished from other risk factors (e.g., wastewater injections). Additionally, most studies lack a non-exposed control group and a reliable baseline, and we know of none that consider the longitudinal effects of exposure.

This lack of information regarding the (long-term) impact of induced seismicity on health is problematic. The occurrence of induced earthquakes is increasingly common across the globe: 1174 projects worldwide report induced seismicity [18]. High-profile cases of induced earthquakes have occurred in Oklahoma, U.S.A., and (on a smaller scale) Lancashire, UK [19, 20]. There are rising concerns regarding the consequences thereof within exposed populations, coupled with calls to policy makers for monitoring and contingency planning [19]. Policy makers need to weigh the wider economic benefits against potential drawbacks for exposed residents [21].

The present work was designed to address the lack of information regarding the long-term impact of induced seismicity for residents: It studies the longitudinal (psychosomatic) health impact of induced seismicity on a group exposed to the consequences of seismicity (damage to housing) versus a control group not exposed to

For peer review only - http://bmjopen.bmj.com/site/about/guidelines.xhtml

these consequences. The study was conducted in the province of Groningen, Netherlands, where conventional gas extraction from the largest gas field in Europe takes place[1]. While the magnitude of seismic events (up to 3.6 Richter) is generally considered 'light', their magnitude has increased over the past 30 years, making this a slow-onset disaster, and their impact is felt well beyond the gas field boundaries. The recurrent earthquakes damage housing in a region not prepared for seismic activity [22] and the governmental response to damage compensation has been considered inadequate [23]

The present study is novel in charting the chronic impact of exposure to damage on health over a time period of almost two years, on a large sample. We tested the following hypotheses: 1. Exposure versus non-exposure will have a negative impact on (psychosomatic) health outcomes. 2. Increases in exposure are related to poorer health outcomes.

METHOD

Setting and exposure

The study was conducted in the province of Groningen, Netherlands, where conventional gas extraction from the largest gas field in Europe takes place. Exposed residents experience rising concerns about physical safety, loss of property value and uncertainty about the future[23, 24].The benefits of extraction flow to the operator (the

[1] See Figure S1 for more information on seismicity in this province

For peer review only - http://bmjopen.bmj.com/site/about/guidelines.xhtml

Netherlands petroleum company, NAM) and the national government, while damage repair and compensation by these entities has been criticized as being inadequate[23].

Seismicity has increased over time. While the magnitude of seismic events (up to 3.6 Richter) is generally considered ‘light’, their impact is felt well beyond the gas field boundaries. Also, multiple factors (limited depth & high rates of occurrence of earthquakes; surface constitution) contribute to considerable damage to housing in a region not prepared for seismic activity[22]. For these reasons, documented damage has proven the most proximal measure of exposure, compared to indicators of seismicity[25].

Sample and recruitment

A stratified random sample was drawn of 25000 residents of the province of Groningen, aged 16 and over, from the official municipal population records which is a complete register of all legal residents. Sampling occurred in areas where damage is reported and from outlying areas where this is not the case. Postal-code areas that were rural and strongly affected by damage were oversampled[2]. Residents received letters with personal login codes and one reminder. Eighteen percent (N=4577) signed up for the study, and later received invitations to all questionnaires. Of these 4577, 86% (3934) filled out the first questionnaire. Baseline equivalence of non-exposed and exposed groups was assessed. Differences between groups were significant but small. Those with multiple damage to homes were slightly younger (r^2 =.014), more highly educated

[2] In the Netherlands, 4-number postal-code areas provide reasonably accurate geographic positioning, whilst preserving anonymity. Data about damage in each area was provided by the institution handling damage claims, the Centrum voor Veilig Wonen.

For peer review only - http://bmjopen.bmj.com/site/about/guidelines.xhtml

(Cramer's V=.062), and more likely to be male (V=.072). The first two characteristics suggest the exposed group might be slightly healthier. We statistically controlled for these characteristics.

Data sources

Procedure

Questionnaires were sent via an email link or by post. A reminder was sent after 2 weeks. Participants (T1: N=3934; T5: N=2150) completed measures at 5 time points during 2 years (T1: February 2016, T2: June 2016; T3: November 2016; T4: April 2017; T5: November 2017; see Table 2).

Study Variables

Exposure to consequences of gas extraction was operationalized in two ways. Chronic physical exposure to ground motion was assessed by the cumulative peak ground acceleration (PGA_{cum}) on the basis of "shakemaps" provided by the Dutch geological survey (KNMI)[3]. Personal exposure to damage due to ground motion was assessed every timepoint by asking participants to indicate how often their home had been damaged (never, once or multiple times)[4].

[3] KNMI calculates shakemaps based on motion sensor readings. For each participant, the PGA of all events modelled by KNMI between 2012 and 2017 was summed, to create an index of exposure to ground motion before and during the study.
[4] See supplementary materials (table S1) for demographic characteristics by level of damage exposure.

For peer review only - http://bmjopen.bmj.com/site/about/guidelines.xhtml

Demographic variables included gender, age and completed education level (categorized into ‘low’ (no, elementary, or pre-vocational education), ‘middle’ (secondary or vocational education), or ‘high’ (higher education) level of education).

(Psychosomatic) health outcomes were assessed at (almost) all time points (Table 2) as follows via:

1. The WHO and Statistics Netherlands recommended validated health survey item assessing *self-rated health [26]* (‘how good is your health in general?’, from ‘very poor’ to ‘excellent’ on a 5 point scale), which is part of the SF-36 [27].

2. *Stress-related health* symptoms were *based on a validated scale of* symptoms of disaster impact [28]. This list of symptoms was shortened by authors (JB, FG, TP): symptoms associated with chronic stress were retained[5]. Consequences of exposure to toxic substances and noise (e.g., hearing problems) were deemed irrelevant for earthquakes and removed. Ten symptoms (stomach problems, heart palpitations, headaches, dizziness/lightheadedness, sensitivity to light/sounds, muscle/joint pains, irritability, memory/concentration problems, insomnia, tiredness) were assessed by asking ‘how often have you experienced the following complaint(s) in the past four weeks’ with response options ‘never, rarely, occasionally, often, most times, continuously’. Aggregate health index scores were computed for stress-related health symptoms, so that individuals have a score of 0 to 100, with 100 representing optimal health. Psychometric properties of the aggregate scale were adequate. Correlations among items ranged from ordinal rho 0.26 to 0.72 (median=0.39). A single factor explained 46% of variance. Scale reliability was good with omega=.90.

[5] Notably, at the level of individuals who suffer these complaints they are referred to as “medically unexplained” because they can have multiple sources, among which is chronic stress.

For peer review only - http://bmjopen.bmj.com/site/about/guidelines.xhtml

3. The five-item validated Mental Health Inventory (MHI-5), part of the Short Form Health Survey (SF-36), measuring general *mental health [27, 29]*. The MHI-5 has a score of 0 to 100. A score of 100 represents optimal mental health.

Data management and Analysis

Analyses controlled for age, gender and education level. Analyses were weighted to correct for sampling effects of age, gender and degree of exposure of postal-code areas[6]. The weights were developed to counteract any potential distortive effect due to age composition, among others (e.g., because younger people were underrepresented, see results section). We report the weighted results. The unweighted results were very similar.

To assess the impact of exposure to gas extraction on health over time, we constructed multilevel conditional growth models on the three health indices with damage to housing as the (between group) predictor[30]. Participants with missing data on the health indices were retained, as multilevel modelling is robust to missingness in estimation of model outcomes.

Models were tested in a step-wise approach, first including control variables (gender, age, level of education) and time. At the next step, physical exposure (PGA_{cum}) was added, followed by earthquake damage at time 1 and the increase of damage since time 1. The final model included the interaction between damage and time. Model fit

[6] As mentioned, we oversampled rural areas as well as the most heavily exposed areas. The geographical weighting was added to control for this overrepresentation.

For peer review only - http://bmjopen.bmj.com/site/about/guidelines.xhtml

was compared to assess which variables best predicted health outcomes. The best fitting models were those including the interaction of damage by time (see Table 3).

To highlight the implications of the findings, we distinguished poor and good health on the basis of health scores, enabling us to compute odds ratios (OR) and relative risk. For mental health we used the conventional criterion of MHI < 60 as cutoff[31]. For perceived health we classified "good" and "outstanding" as good health and all other scale points as poor (conform international convention). For symptoms we devised our own cutoff based on distributional characteristics combined with content criteria: A classification of < 60 as poor health resulted in 9% of the unaffected population being classified as such. Odds ratios were calculated in weighted models, controlling for age, education and gender.

Public involvement

The research setup (design and outcome measures) was discussed with an advisory board consisting of institutions (e.g., local municipalities) and representatives of the public (e.g., action groups). The present work has been disseminated in a public report.

RESULTS

Sample characteristics

There were no significant fluctuations in sample composition over time in terms of gender, education level and damage to own housing (see Table 2). Young respondents were underrepresented. There was attrition during the study. Dropout characteristics revealed no differences between exposed vs. control groups and no association between dropout and health. Analyses showed no indications that attrition

For peer review only - http://bmjopen.bmj.com/site/about/guidelines.xhtml
For peer review only

influenced any of the effects reported below. Over time, the average age of participants increased, as young people tended to have a higher likelihood of dropout. It is important to note that additional analyses found no significant interaction effect between age and exposure, suggesting that the effects of exposure were age-independent. Because the sample was not entirely representative and attrition relatively high, we carefully checked the potential consequences thereof and found no indications this influenced results.

Regarding levels of exposure, we know from the damage claims register[7] that the rates of exposure vary substantially within the region: in central areas up to 100% of homes have reported damage at least once. Outside these areas, there is progressively less damage. A substantial part of the province has (nearly) no damage. Average levels of damage are closely associated with ground motion[25]: In postal-code areas where 0% damage was reported until January 2016, there was hardly any exposure to ground motion (total ground motion PGA_{cum}=.07 mm/s^2). Only 3% of the sample located in this area suspected having damage due to earthquakes. In the areas where up to 20% damage was previously reported, total ground motion was somewhat higher PGA_{cum}=0.64 mm/s$^{2)}$) and more people, 26% of the sample, indicated suspecting they have damage. And in the areas where 20% to 100% had reported damage, ground motion was considerably higher, PGA_{cum}=4.13 mm/s^2 and high percentage of our sample, 83%, suspected having damage.

The impact of exposure to gas extraction on health over time

[7] Provided by the institution handling damage claims, the Centrum voor Veilig Wonen

For peer review only - http://bmjopen.bmj.com/site/about/guidelines.xhtml

The analyses of conditional growth models on self-rated health, stress-related health problems and mental health showed consistent results across all three indicators. Table 3 shows the final results for all variables.

Important to note is that, after including control variables in step 1, there was a significant effect of exposure to physical ground motion (PGA_{cum}) on all three health indicators: more ground motion was associated with poorer health. The effect of time was also significant: over time, health deteriorated.

In step 2, we included damage to housing. Having damage once had no significant effect on any of the health indicators. Only participants with multiple damages experienced negative health consequences.

The effects of ground motion were suppressed by the larger effects of exposure to multiple damage on all health indicators (p's<.01). The suppression occurs because damage and total ground motion are strongly correlated. It does not mean that the association between exposure to ground motion and health should be disregarded: There might, for example, be some health effects of "peak exposure" to strong ground motion in the weeks or months after an earthquake. The current analysis does not address such peak exposure effects because it only assesses average impact on health over the entire two-year period and gradual changes in health over time.

In step 3, the significant ‘multiple damage (vs no damage) X time’ interaction reveals that exposure to multiple damages is associated with a deterioration of health over time. The inclusion of this interaction variable improved model fit.

To interpret these effects of exposure to damage and assess their magnitude, we calculated odds ratios for health measures at every time point, as well as the average

For peer review only - http://bmjopen.bmj.com/site/about/guidelines.xhtml

impact of exposure over time (Table 4). Inhabitants exposed to damage once are only marginally (and not significantly) affected compared with a no-damage control group (averaged odds ratios range from 1.10 to 1.20). Those exposed to damage multiple times are more likely to report poor self-rated health (OR = 1.64, with a 95% confidence interval of 1.31;2.04), more stress-related health symptoms (OR = 2.52 [1.89;3.38]) and less good mental health (OR = 1.83 [1.40;2.39]) than those without damage. This indicates that damage has a considerable impact on participants' health[8].

The table also suggests that differences between groups increase over time. Odds ratios for the difference between those with multiple damage and no damage are considerably higher 21 months after first measurement for self-rated health (OR = 2.00 [1.57;2.55]), mental health (OR = 2.38 [1.78;3.21]) and stress related health symptoms (OR = 3.36 [2.45;4.68]). In terms of relative risk, this means that those whose homes have multiple damage at T5 are 1.60[1.37;1.86] times more likely to report poor health, 2.11 [1.63;2.74] times more likely to report negative mental health and 2.84 [2.14;3.76] times more at risk of elevated levels of stress related health symptoms.

We also compared the weighted means of the ORs of control variables that are known correlates of health (age, gender, level of education) to the effect of damage (Figure 1). Across the three health measures, effect sizes (ORs) of damage are slightly larger than those of education (for a high versus low level of education, the average odds ratios over time are .53 [.41;.68] for self-rated health; OR = .58 [.42;.81] for mental health; and OR = .56 [.41;.79] for stress-related health symptoms).

---

[8] We also investigated whether women's health is affected differently by this stressor than men's, but as evidenced in Table S2 in the supplementary materials, this is not the case.

For peer review only - http://bmjopen.bmj.com/site/about/guidelines.xhtml

DISCUSSION

Natural and induced seismicity can have negative consequences for local populations due to (acute or accumulated) health threats and irreversible changes to the living environment. Yet, so far studies have not assessed the accumulated impact of (the consequences of) induced seismicity on (psychosomatic) health *over time*. Moreover, most studies lack a non-exposure control group. The present study aimed to address these shortcomings by studying the impact of exposure to gas extraction (and subsequent damage to housing), compared to a no-exposure control group, on health over a time period of 21 months. Our study provides strong indications that exposure to negative side-effects of induced seismicity (e.g., damage to people's homes) constitutes an increasing health risk over time: We found that those who self-reported having multiple damages to housing experienced more negative health consequences than those without damage. Moreover, these effects increased over time. Results showed that chronic physical exposure to ground motion (assessed objectively) was also related to health, although less strongly than reporting multiple personal damages .

To our knowledge, this is the only study of the long-term impact of induced seismicity on health. Therefore, we can only compare our results with the long-term impacts of very different types of disaster - limiting comparability. For one, the Chernobyl nuclear disaster: Study participants lived in a seriously contaminated area approximately 50 miles from Chernobyl. 6.5 years post disaster, inhabitants were twice as likely to have negative self-rated health (OR:2.25[1.96-2.58]) and psychological distress (OR:1.93[1.69-2.22]), compared to a non-exposed control group[32]. Chernobyl clearly constitutes a very different type of disaster and health risk (radiation

For peer review only - http://bmjopen.bmj.com/site/about/guidelines.xhtml

exposure). The Brisbane floods were also very different in many respects (e.g., sudden disaster onset; deaths) but with some comparable outcomes, such as considerable damage to homes. 6-7 months post-disaster, those exposed to flooding were twice as likely to report psychological distress compared to the non-exposed[33]. It appears that the health impact of these very different and in many ways more 'acute' disasters are, in terms of effect size, somewhat comparable to the health impact of the more chronic exposure to induced earthquakes caused by gas extraction. One potential reason for the comparable effect sizes, is that our study focused not just on the environmental effect (e.g., amount of total ground motion) but zoomed in on the subgroup who were severely affected because they had multiple instances of damage to their own home. We further speculate that the man-made nature of the hazard (the fact that earthquakes are induced) may also enhance the impact on the population.

The present work also provides first time insights into the development of (psychosomatic) health symptoms in response to chronic disaster. In the area of acute disaster response, studies on longitudinal health impact reveal that distress decreases over time[34, 35], implying recovery of victims. Yet looking at discussions comparing chronic man-made (technological) disasters to acute natural disasters, we see the present context shares elements identified as reasons for potential long-term health impact of technological disasters: A strong element of culpability in causing disaster, concerns about damage compensation after disaster and uncertainty regarding when disaster impact will end ("the book is never closed"; p.148, [12]; [36-38]. In line with this work, our findings suggest that for chronic disasters/hazards, negative effects can accumulate over time, presumably because the recurrent threat and poor crisis

For peer review only - http://bmjopen.bmj.com/site/about/guidelines.xhtml

response leads to an accumulation of stress.

Limitations

A potential limitation of this sample could be concerns about its representativeness: For one, attrition was 45.3% over time and younger respondents were somewhat underrepresented. However, attrition was no different for the exposed and non-exposed groups, was unrelated to health outcomes, and all further analyses suggest that neither attrition nor sample characteristics had any substantial influence on results and conclusions drawn. Secondly, there might be an influence of confounding variables. Yet we believe effect sizes are robust: 1. The exposed and control groups were very similar regarding key population and geographical characteristics 2. Follow-up analyses revealed no interactions between any of the population characteristics and the effects of exposure.

Thirdly, responses could have been biased because participants knew the survey was about the social impact of gas extraction. It is relevant here that an ‘objective’ exposure measure (peak ground acceleration) revealed comparable health outcomes to self-reported exposure. Moreover, analyses on a cross-sectional representative sample of residents (N=16340) in the 2016 health monitor of Statistics Netherlands, the National Institute for Public Health and public health services found comparable results. In this survey, the study intent was not clear.

One of the three health measures included, stress-related health symptoms, was an adaptation of a previously validated symptoms list[39], shortened for this specific study. Although the shortened version was not previously validated, it was psychometrically sound. Also, patterns are comparable across health measures, two of

For peer review only

which are validated.

One of our exposure measures is self-reported damage. It is possible that damage is perceived differently depending on people's health status. Importantly, physical exposure to ground motion was associated with significant health effects. But effects of damage were stronger. This could be because damage is a more precise and proximate indicator of how individuals are affected by exposure, but also because of recursive effects of (mental) health on perceived damage.

An important issue is generalizability: Is the situation in Groningen comparable to other areas with induced seismicity (e.g., fracking, wastewater injections)? We can only make reasoned inferences. Induced earthquakes are relatively common in energy projects which involve injection[40]. A priori, similar health consequences could occur in all sites in which populations are affected by induced earthquakes. Moreover, the vulnerability of people exposed to seismicity is likely influenced by similar factors: negative consequences are man-made and involve safety, health and social risks [12, 13]. In sum, although more research on the impact of induced seismicity is needed [41], we suggest effects are likely to generalize beyond the Groningen case.

Practical implications

The consequences of induced seismicity pose challenges to decision-makers. Benefits to the public good need to be balanced against the welfare of local populations [21]. As projects involving induced seismicity rapidly grow, governments and businesses face decisions whether to invest and how to manage risks. Our work provides a case study of what occurs if seismicity is not kept in check. It can increase awareness of the

For peer review only

vulnerability of exposed populations and provide important input for future decision making, monitoring and contingency planning.

Conclusion

Recent years have seen a rise in induced seismicity. Little is known about the (longitudinal) impact thereof on (psychosomatic) health. The present study is the first to our knowledge evidencing the long-term impact of induced seismicity on health.

For peer review only

For peer review only - http://bmjopen.bmj.com/site/about/guidelines.xhtml

Funding: This study was funded by the National Coordinator Groningen. Award/grant number: not applicable.

Contributorship: KS, BK, JR, JB, FO, FG and TP contributed to the research questions and study design. JR and FO contributed to data collection. BK and TP conducted statistical analyses. KS, BK and TP interpreted the results and wrote the initial draft of the manuscript. All authors commented on the final draft of the manuscript. BK and TP had full access to the data in the study and can take responsibility for the integrity of the data and the accuracy of the data analysis.

Conflict of Interest: The authors declare that they have no conflict of interest.

Ethical approval: All procedures performed in studies involving human participants were in accordance with the ethical standards of the ethical board of the department of psychology of the University of Groningen, The Netherlands (research code ppo-015-085).

Informed consent: Informed consent was obtained from all individual participants included in the study.

Data sharing statement: Data collected for this study are not available in order to guarantee anonimity of participants.

For peer review only

Table 1.

List of definitions

Conventional gas extraction	Extraction through drilling in deep subsoil reservoirs without the injection of chemical liquids.
Fracking	A stimulation technique in which a rock is fractured by a pressured liquid in order to extract oil or gas from wells.
Induced seismicity	Seismic events that are a result of human activity.
Natural seismicity	Seismic events that have a natural cause (e.g., volcanic eruption).
Peak ground acceleration	Measure of the maximum increase in ground motion during an earthquake, recorded by a ground motion sensor.
Psychosomatic health	Health outcomes involving both mind and body.
Richter scale	Measure of strength of earthquakes with a logarithmic scale.
Shale gas	A natural gas that is trapped in fine grained sediment in rock.
Unconventional gas extraction	Gas reservoirs that require a special stimulation technique to extract gas (e.g., by injecting large quantities of fluids underground).

For peer review only

Table 2

Demographic characteristics of participants participating in separate measurements: total number of participants participating in that measurement, decline of number of participants participating as compared to the number of participants participating at T1, mean age, distribution of level of education, distribution of personal exposure to damage due to gas extraction, distribution of gender, and amount of participants that completed the three health measures in that measurement. Netherlands 2016-2017

		T1 Feb '16	T2 June '16	T3 Nov '16	T4 Apr '17	T5 Nov '17
Total N		3934	3153	2638	2351	2150
Attrition (compared to T1)		-	19.9%	32.9%	40.2%	45.3%
Age (mean)		56.54	57.74	57.72	58.90	59.98
Level of education (N)	Low	968 (24.6%)	772 (24.5%)	616 (23.4%)	589 (25.1%)	535 (24.9%)
	Middle	1252 (31.8%)	970 (30.8%)	815 (30.9%)	713 (30.3%)	639 (29.7%)
	High	1533 (39.0%)	1238 (39.3%)	1068 (40.5%)	944 (40.2%)	852 (39.6%)
Gender (N)	Male	1967 (50.0%)	1547 (49.1%)	1306 (49.5%)	1182 (50.3%)	1068 (49.7%)
	Female	1849 (47.0%)	1480 (46.9%)	1231 (46.7%)	1097 (46.7%)	990 (46.0%)
Exposure to damage (N)	None	1477 (37.5%)	1204 (38.2%)	1027 (38.9%)	910 (38.7%)	846 (39.3%)
	One time	913 (23.2%)	626 (19.9%)	554 (21.0%)	505 (21.5%)	459 (21.3%)
	Multiple	1057 (26.9%)	1055 (33.5%)	940 (35.6%)	775 (33.0%)	736 (34.2%)
Perceived health (N)		3821 (97.1%)	-	2540 (96.3%)	2206 (93.8%)	2059 (95.8%)
Stress related health symptoms (N)		3767 (95.8%)	-	2533 (96.0%)	2206 (93.8%)	2045 (95.1%)
Mental health (N)		3711 (94.3%)	2819 (89.4%)	2501 (94.8%)	2179 (92.7%)	2021 (94.0%)

For peer review only

Table 3
Results of multilevel conditional growth models: *Unstandardized parameter estimates and standard errors for the association between time, damage, and the interaction between time and damage on perceived health, stress-related health symptoms, and mental health – adjusted for gender, age, level of education and ground motion (cumulative PGA). Netherlands 2016-2017.*

	Perceived health	Stress-related health symptoms[1]	Mental health
Gender	-0.05*	-5.40***	-2.68***
	(0.02)	(0.49)	(0.46)
Age	-0.01***	-0.02	0.07***
	(0.001)	(0.02)	(0.02)
Level of education (middle)	0.08*	0.61	1.01
	(0.03)	(0.67)	(0.62)
Level of education (high)	0.24***	3.02***	2.94***
	(0.03)	(0.63)	(0.59)
Cumulative PGA	-0.001	0.03	-0.01
	(0.004)	(0.09)	(0.08)
Time	-0.01	-0.25*	-0.49***
	(0.01)	(0.13)	(0.15)
Damage (one time)	-0.01	-0.46	-0.27
	(0.03)	(0.75)	(0.63)
Damage (multiple)	-0.12***	-4.31***	-3.35***
	(0.03)	(0.76)	(0.65)
Time * Damage (one time)	-0.02	-0.13	-0.07
	(0.01)	(0.20)	(0.24)
Time * Damage (multiple)	-0.03***	-0.45*	-0.60**
	(0.01)	(0.19)	(0.23)
Constant	3.86***	80.19***	77.78***
	(0.03)	(0.67)	(0.60)
Observations	10,256	9,100	9,686
Log Likelihood	-10,104.58	-36,205.01	-38,020.51
Akaike Inf. Crit.	20,239.16	72,440.02	76,071.02
Bayesian Inf. Crit.	20,347.69	72,546.76	76,178.69

Note. $^{*}p<0.05$; $^{**}p<0.01$; $^{***}p<0.001$

1. Stress-related health symptoms were reverse-coded such that higher levels indicate less stress

For peer review only

Table 4

Proportion of participants who have poor health and OR of participants who have poor health with damage (compared to those with no damage) across measurements, with 95% confidence intervals – adjusted for age, gender and level of education. Netherlands 2016-2017

Measurement	Damage	Percentage poor health	Odds ratio
Self-rated health			
T1	None	22.2% [19.9%;24.5%]	-
	One time	22.5% [19.6%;25.4%]	1.02 [0.82;1.26]
	Multiple	25.6% [22.7%;28.4%]	1.21 [0.99;1.47]
T3	None	21.6% [18.8%;24.3%]	-
	One time	24.4% [20.5%;28.3%]	1.17 [0.90;1.53]
	Multiple	32.4% [29.2%;35.7%]	1.75 [1.41;2.18]
T4	None	21.3% [18.4%;24.2%]	-
	One time	30.0% [25.7%;34.4%]	1.60 [1.22;2.09]
	Multiple	35.5% [31.8%;39.2%]	2.06 [1.63;2.61]
T5	None	23.6% [20.3%;26.9%]	-
	One time	27.5% [22.9%;32.1%]	1.23 [0.92;1.65]
	Multiple	38.0% [34.0%;42.0%]	2.00 [1.57;2.55]
Weighted average	None	22.1% [19.4%;24.9%]	-
	One time	25.6% [21.8%;29.4%]	1.20 [0.93;1.55]
	Multiple	31.8% [28.5%;35.2%]	1.64 [1.31;2.04]
Symptoms			
T1	None	9.2% [7.7%;10.8%]	-
	One time	10.0% [7.9%;12.1%]	1.09 [0.81;1.47]
	Multiple	17.3% [14.9%;19.7%]	2.08 [1.62;2.68]
T3	None	7.1% [5.5%;8.8%]	-
	One time	6.5% [4.3%;8.6%]	0.90 [0.58;1.37]
	Multiple	13.7% [11.4%;16.1%]	2.09 [1.55;2.85]
T4	None	8.1% [6.2%;10.0%]	-
	One time	9.4% [6.7%;12.1%]	1.18 [0.78;1.75]
	Multiple	21.8% [18.7%;25.0%]	3.24 [2.40;4.42]
T5	None	7.1% [5.3%;9.0%]	-
	One time	9.1% [6.3%;11.9%]	1.30 [0.84;1.99]
	Multiple	20.3% [17.0%;23.5%]	3.36 [2.45;4.68]
Weighted average	None	8.0% [6.4%;9.8%]	-
	One time	8.8% [6.5%;11.2%]	1.10 [0.75;1.60]
	Multiple	18.0% [15.3%;20.7%]	2.52 [1.89;3.38]
Mental health			

For peer review only

For peer review only - http://bmjopen.bmj.com/site/about/guidelines.xhtml

T1	None	8.5% [7.0%;10.0%]	-
	One time	9.0% [7.0%;10.9%]	1.06 [0.78;1.43]
	Multiple	12.4% [10.3%;14.5%]	1.53 [1.18;1.98]
T2	None	8.5% [6.8%;10.2%]	-
	One time	9.2% [6.9%;11.6%]	1.09 [0.77;1.54]
	Multiple	18.1% [15.6%;20.7%]	2.40 [1.86;3.13]
T3	None	11.1% [9.0%;13.2%]	-
	One time	12.0% [9.2%;14.9%]	1.10 [0.79;1.51]
	Multiple	14.5% [12.1%;16.9%]	1.36 [1.04;1.77]
T4	None	11.9% [9.6%;14.1%]	-
	One time	11.8% [8.9%;14.7%]	0.99 [0.71;1.38]
	Multiple	20.3% [17.2%;23.4%]	1.9 [1.46;2.47]
T5	None	9.0% [6.9%;11.1%]	-
	One time	12.5% [9.3%;15.7%]	1.44 [0.99;2.07]
	Multiple	19.1% [15.9%;22.2%]	2.38 [1.78;3.21]
Weighted average	None	9.7% [7.8%;11.5%]	-
	One time	10.6% [8.1%;13.2%]	1.11 [0.80;1.55]
	Multiple	16.4% [13.8%;19.0%]	1.83 [1.40;2.39]

Note. Scores were categorised as low health as follows: 1) very poor, poor, or fair perceived health; 2) a score below 60 for stress related health symptoms, and 3) a score below 60 for mental health.

For peer review only - http://bmjopen.bmj.com/site/about/guidelines.xhtml

For peer review only

REFERENCES

1 Konkel L. In the Neighborhood of 18 Million: Estimating How Many People Live Near Oil and Gas Wells, *Environ Health Perspect* 2017;125:UNSP 124003 doi:10.1289/EHP2553 [published Online First: DEC].

2 U.S. Energy Information Administration. United States Shale gas reserves per state. 2017;2019.

3 Dai W, Chen L, Lai Z, et al. The incidence of post-traumatic stress disorder among survivors after earthquakes: A systematic review and meta-analysis, *BMC Psychiatry* 2016;16.

4 Neria Y, Nandi A, Galea S. Post-traumatic stress disorder following disasters: A systematic review, *Psychol Med* 2008;38:467-80 doi:10.1017/S0033291707001353.

5 Beaglehole B, Mulder RT, Frampton CM, et al. Psychological distress and psychiatric disorder after natural disasters: systematic review and meta-analysis, *The British Journal of Psychiatry* 2018;213:716-22.

6 Goldmann E, Galea S. Mental health consequences of disasters, *Annu Rev Public Health* 2014;35:169-83.

7 Galea S, Nandi A, Vlahov D. The epidemiology of post-traumatic stress disorder after disasters, *Epidemiol Rev* 2005;27:78-91.

8 Norris FH, Friedman MJ, Watson PJ. 60,000 disaster victims speak: Part II. Summary and implications of the disaster mental health research, *Psychiatry: Interpersonal and biological processes* 2002;65:240-60.

9 Rubonis AV, Bickman L. Psychological impairment in the wake of disaster: The disaster–psychopathology relationship. *Psychol Bull* 1991;109:384.

10 Keranen KM, Weingarten M. Induced Seismicity, *Annual Review of Earth and Planetary Sciences, Vol 46* 2018;46:149-74 doi:10.1146/annurev-earth-082517-010054.

11 van der Elst, Nicholas J., Page MT, Weiser DA, et al. Induced earthquake magnitudes are as large as (statistically) expected, *Journal of Geophysical Research-Solid Earth* 2016;121:4575-90 doi:10.1002/2016JB012818 [published

For peer review only - http://bmjopen.bmj.com/site/about/guidelines.xhtml

Online First: JUN].

12 Couch SR, Kroll-Smith JS. The chronic technical disaster: Toward a social scientific perspective, *Social Science Quarterly* 1985;66:564.

13 Sangaramoorthy T, Jamison AM, Boyle MD, et al. Place-based perceptions of the impacts of fracking along the Marcellus Shale, *Soc Sci Med* 2016;151:27-37.

14 Rabinowitz PM, Slizovskiy IB, Lamers V, et al. Proximity to Natural Gas Wells and Reported Health Status: Results of a Household Survey in Washington County, Pennsylvania, *Environ Health Perspect* 2015;123:21-6 doi:10.1289/ehp.1307732.

15 Steinzor N, Subra W, Sumi L. Investigating links between shale gas development and health impacts through a community survey project in Pennsylvania. *New solutions : a journal of environmental and occupational health policy : NS* 2013;23:55-83 doi:10.2190/NS.23.1.e.

16 Tustin AW, Hirsch AG, Rasmussen SG, et al. Associations between Unconventional Natural Gas Development and Nasal and Sinus, Migraine Headache, and Fatigue Symptoms in Pennsylvania, *Environ Health Perspect* 2017;125:189-97 doi:10.1289/EHP281.

17 Weinberger B, Greiner LH, Walleigh L, et al. Health symptoms in residents living near shale gas activity: A retrospective record review from the Environmental Health Project. *Preventive medicine reports* 2017;8:112-5 doi:10.1016/j.pmedr.2017.09.002.

18 Wilson MP, Foulger GR, Gluyas JG, et al. HiQuake: The human-induced earthquake database, *Seismol Res Lett* 2017;88:1560-5.

19 Aczel MR, Makuch KE. Shale, Quakes, and High Stakes: Regulating Fracking-Induced Seismicity in Oklahoma, USA and Lancashire, UK, *Case Studies in the Environment* 2019.

20 Keranen KM, Weingarten M, Abers GA, et al. Sharp increase in central Oklahoma seismicity since 2008 induced by massive wastewater injection, *Science* 2014;345:448-51 doi:10.1126/science.1255802 [published Online First: JUL 25].

21 Fleming RC, Reins L. Shale gas extraction, precaution and prevention: A conversation on regulatory responses, *Energy Research & Social Science* 2016;20:131-41 doi:10.1016/j.erss.2016.05.013 [published Online First: OCT].

For peer review only

For peer review only - http://bmjopen.bmj.com/site/about/guidelines.xhtml

22 Vlek C. Rise and reduction of induced earthquakes in the Groningen gas field, 1991-2018: statistical trends, social impacts, and policy change, *Environ Earth Sci* 2019;78:59 doi:10.1007/s12665-019-8051-4 [published Online First: FEB].

23 Dutch Safety Board. Earthquake risks in Groningen. Research concerning the role population safety played in decision making procedures about gas extraction (1959-2014) , 2015.

24 Postmes T, Stroebe K, Richardson J, et al. Consequences of ground motion for the Groningen population: Experienced safety, health and future perspective 2016-2017. *University of Groningen* 2018.

25 Geurts C, Steenbergen R. Note for the Dutch State Supervision of Mines: The relation between Peak Ground Acceleration and the risk of damage for induced earthquakes in Groningen. *The Netherlands Organisation for Applied Scientific Research* 2016.

26 Pappas G. Health interview surveys: Towards international harmonization of methods and instruments - deBruin,A, Picavet,HSJ, Nossikoy,A, *Soc Sci Med* 1997;44:1431-2 doi:10.1016/S0277-9536(97)84078-6 [published Online First: MAY].

27 Ware JE, Gandek B, IQOLA Project. Overview of the SF-36 Health Survey and the International Quality of Life Assessment (IQOLA) Project, *J Clin Epidemiol* 1998;51:903-12 doi:10.1016/S0895-4356(98)00081-X [published Online First: NOV].

28 Van den Berg B, Yzermans CJ, Van der Velden, Peter G., et al. Risk Factors for Unexplained Symptoms After a Disaster: A Five-Year Longitudinal Study in General Practice, *Psychosomatics* 2009;50:69-77 doi:10.1176/appi.psy.50.1.69.

29 Berwick DM, Murphy JM, Goldman PA, et al. Performance of a five-item mental health screening test. *Med Care* 1991.

30 Raudenbush SW, Bryk AS. Hierarchical linear models: Applications and data analysis methods: Sage 2002.

31 Rumpf H, Meyer C, Hapke U, et al. Screening for mental health: validity of the MHI-5 using DSM-IV Axis I psychiatric disorders as gold standard, *Psychiatry Res* 2001;105:243-53.

For peer review only - http://bmjopen.bmj.com/site/about/guidelines.xhtml

32 Havenaar J, Rumyantzeva G, Kasyanenko A, et al. Health effects of the Chernobyl disaster: Illness or illness behavior? A comparative general health survey in two former Soviet regions, *Environ Health Perspect* 1997;105:1533-7 doi:10.2307/3433666 [published Online First: DEC].

33 Alderman K, Turner LR, Tong S. Assessment of the Health Impacts of the 2011 Summer Floods in Brisbane, *Dis Med Public Health Prep* 2013;7:380-6 doi:10.1017/dmp.2013.42 [published Online First: AUG].

34 Lowell A, Suarez-Jimenez B, Helpman L, et al. 9/11-related PTSD among highly exposed populations: a systematic review 15 years after the attack, *Psychol Med* 2018;48:537-53 doi:10.1017/S0033291717002033 [published Online First: MAR].

35 van der Velden, Peter G., Wong A, Boshuizen HC, et al. Persistent mental health disturbances during the 10years after a disaster: Four-wave longitudinal comparative study, *Psychiatry Clin Neurosci* 2013;67:110-8 doi:10.1111/pcn.12022 [published Online First: FEB].

36 Baum A. Implications of psychological research on stress and technological accidents. *Am Psychol* 1993;48:665.

37 Picou JS. The “talking circle” as sociological practice: Cultural transformation of chronic disaster impacts, *Sociological Practice* 2000;2:77-97.

38 Picou JS, Marshall BK, Gill DA. Disaster, litigation, and the corrosive community, *Social forces* 2004;82:1493-522.

39 van den Berg B, Grievink L, Yzermans J, et al. Medically unexplained physical symptoms in the aftermath of disasters, *Epidemiol Rev* 2005;27:92-106 doi:10.1093/epirev/mxi001.

40 Ellsworth WL. Injection-induced earthquakes, *Science* 2013;341:1225942.

41 Hirsch JK, Smalley KB, Selby-Nelson EM, et al. Psychosocial Impact of Fracking: a Review of the Literature on the Mental Health Consequences of Hydraulic Fracturing, *International Journal of Mental Health and Addiction* 2018;16:1-15 doi:10.1007/s11469-017-9792-5 [published Online First: FEB].

For peer review only

For peer review only - http://bmjopen.bmj.com/site/about/guidelines.xhtml

Figure 1. Weighted average odds ratios.

For peer review only

For peer review only - http://bmjopen.bmj.com/site/about/guidelines.xhtml

Figure 1. Weighted average odds ratios.

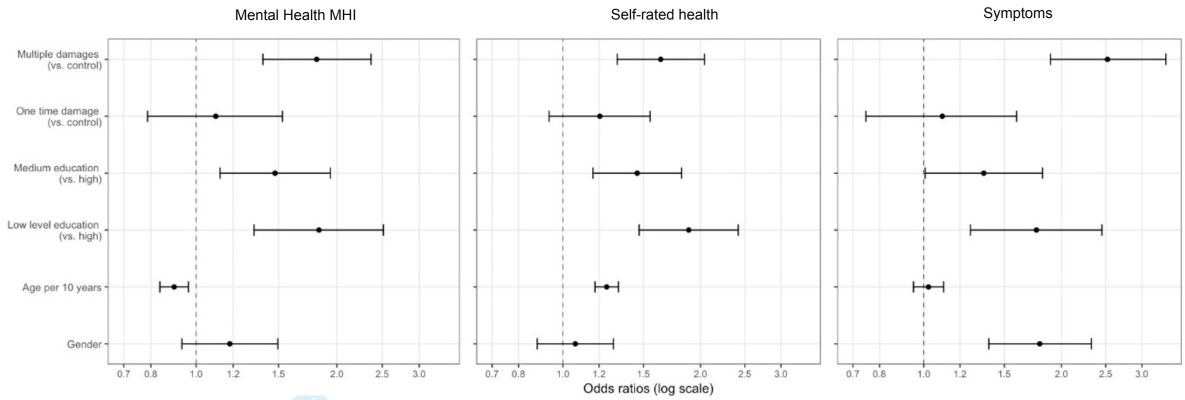

For peer review only

Supplementary materials

Seismicity in the Groningen gasfield

Figure S1: Number of earthquakes and volume of gas production in the Groningen gasfield:

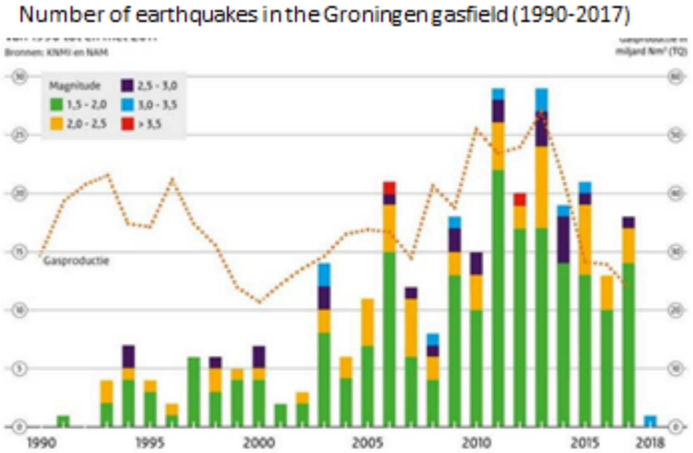

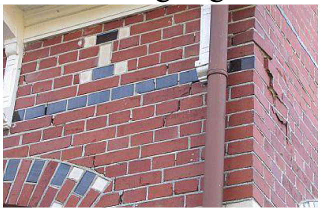

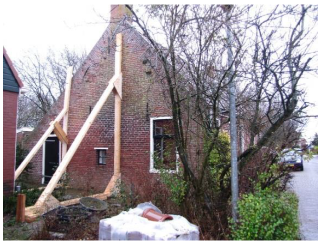

The volume of gas production in the subsoil of Groningen is presented as a dotted line in Figure 2. The number of earthquakes magnitude 2 or higher increased from 2003 onwards. The earthquake of August 2012 in the village of Huizinge (magnitude 3,6 on Richter scale) was a landmark event that caused unrest and great concern among the population in the province of Groningen. It was the heaviest earthquake measured in the province. The magnitude of earthquakes is recorded by the Dutch national borehole network, the regional accelerometer network and all additional seismic stations in the south of the Netherlands. European seismic stations reported the event at epicentral distances up to 800 km (Dost and Kraaipoel, 2012).

410,000 residents of the province of Groningen are exposed to these induced earthquakes: they live in a postcode area where damage has been recognized by the oil company responsible (NAM, a joint venture of Shell and Exxon). Of these, 134,363 adults report having damage to their property (Postmes et al., 2017). Of these 68,343 report having damage multiple times.
Looking worldwide, there are 1174 locations in which induced seismicity is taking place. 11% of seismicity is due to conventional oil or gas extraction (see inducedearthquakes.org/)

References

Dost B and Kraaipoel D. The August 16, 2012 earthquake near Huizinge in Groningen province. Royal Dutch Meteorological Institute, 2013.

Postmes, T., Stroebe, K., Richardson, J., LeKander, B., Oldersma, F., Broer, J. & Greven, F. (2018). Gevolgen van bodembeweging voor Groningers: Ervaren veiligheid, gezondheid en toekomstperspectief 2016-2017. Groningen: Heymans Instituut, Rijksuniversiteit Groningen.

review only

Table S1

Demographic characteristics of participants completing separate measurements per level of damage: total sample size, mean age, distribution of level of education, distribution of level of damage, distribution of gender, and amount of participants that completed the health measures. Netherlands 2016-2017

Damage to house at T1			T1 Feb '16	T2 June '16	T3 Nov '16	T4 Apr '17	T5 Nov '17
None	Total N		1477	1166	968	886	801
	Age (mean)		57.67	59.13	59.13	60.28	61.48
	Level of education (N)	Low	430	340	266	267	238
		Middle	460	351	290	255	226
		High	562	453	396	349	324
	Gender (N)	Male	794	621	515	471	427
		Female	683	545	453	415	374
	Perceived health (N)		1467	-	934	835	784
	Stress related health symptoms (N)		1452	-	937	836	780
	Mental health (N)		1432	1048	920	828	769
One time	Total N		913	730	608	559	490
	Age (mean)		58.32	58.87	58.81	60.06	60.86
	Level of education (N)	Low	237	191	155	143	131
		Middle	295	235	195	182	159
		High	363	295	250	227	193
	Gender (N)	Male	505	398	345	323	279
		Female	407	332	263	236	211
	Perceived health (N)		907	-	587	521	464
	Stress related health symptoms (N)		894	-	584	522	463
	Mental health (N)		895	666	581	517	456
Multiple	Total N		1057	825	704	609	558
	Age (M)		54.06	55.57	55.60	56.71	57.70
	Level of education (N)	Low	215	168	133	120	110
		Middle	381	289	246	213	188
		High	445	356	315	268	253
	Gender (N)	Male	493	385	323	284	269
		Female	563	440	381	325	289
	Perceived health (N)		1048	-	683	578	537
	Stress related health symptoms (N)		1041	-	675	577	530
	Mental health (N)		1018	739	674	570	528

For peer review only

Table S2

Unstandardized regression parameter estimates and standard errors for the association between time, damage, and the interaction between time and damage on perceived health, stress-related health symptoms, and mental health, and the interaction between gender and damage on perceived health, stress-related health symptoms, and mental health – adjusted for gender, age, level of education and ground motion (cumulative PGA). Netherlands 2016-2017.

	Perceived health	Stress-related health symptoms[1]	Mental health
Gender	-0.07	-5.08***	-2.81***
	(0.04)	(0.75)	(0.70)
Age	-0.01***	-0.02	0.07***
	(0.001)	(0.02)	(0.02)
Level of education (middle)	0.08*	0.62	0.99
	(0.03)	(0.67)	(0.62)
Level of education (high)	0.23***	3.03***	2.92***
	(0.03)	(0.63)	(0.59)
Cumulative PGA	-0.001	0.03	-0.01
	(0.004)	(0.09)	(0.08)
Time	-0.01	-0.25*	-0.49***
	(0.01)	(0.13)	(0.15)
Damage (one time)	-0.02	-0.19	-0.05
	(0.04)	(0.93)	(0.81)
Damage (multiple)	-0.14**	-4.04***	-3.79***
	(0.04)	(0.95)	(0.84)
Time * Damage (one time)	-0.02	-0.13	-0.07
	(0.01)	(0.20)	(0.24)
Time * Damage (multiple)	-0.03***	-0.45*	-0.60**
	(0.01)	(0.19)	(0.23)
Gender * Damage (one time)	0.03	-0.58	-0.51
	(0.06)	(1.21)	(1.13)
Gender * Damage (multiple)	0.04	-0.55	0.85
	(0.05)	(1.15)	(1.07)
Constant	3.87***	80.04***	77.86***
	(0.03)	(0.73)	(0.65)
Observations	10,256	9,100	9,686
Log Likelihood	-10,104.34	-36,204.85	-38,019.84
Akaike Inf. Crit.	20,242.68	72,443.70	76,073.68
Bayesian Inf. Crit.	20,365.69	72,564.67	76,195.71

Note. *p<0.05; **p<0.01; ***p<0.001

1. Stress-related health symptoms were reverse-coded such that higher levels indicate less stress

STROBE Statement—checklist of items that should be included in reports of observational studies

	Item No.	Recommendation	Page No.	Relevant text from manuscript
**Title and abstract**	1	(*a*) Indicate the study's design with a commonly used term in the title or the abstract	1/2	Title is mentioned on page 1
		(*b*) Provide in the abstract an informative and balanced summary of what was done and what was found	2	
**Introduction**				
Background/rationale	2	Explain the scientific background and rationale for the investigation being reported	4/5	
Objectives	3	State specific objectives, including any prespecified hypotheses	5/6	The present work was designed to address the lack of information regarding the long-term impact of induced seismicity for residents: It studies the longitudinal (psychosomatic) health impact of induced seismicity on a group exposed to the consequences of seismicity (damage to housing) versus a control group not exposed to these consequences." "The present study is novel in charting the chronic impact of exposure to damage on health over a time period of almost two years, on a large sample. We tested the following hypotheses: 1. Exposure versus

For peer review only - http://bmjopen.bmj.com/site/about/guidelines.xhtml

For peer review only

				non-exposure will have a negative impact on (psychosomatic) health outcomes. 2. Increases in exposure are related to poorer health outcomes."
**Methods**				
Study design	4	Present key elements of study design early in the paper	5-8	
Setting	5	Describe the setting, locations, and relevant dates, including periods of recruitment, exposure, follow-up, and data collection	6-8	"A stratified random sample was drawn of 25000 residents of the province of Groningen, aged 16 and over, from the official municipal population records which is a complete register of all legal residents. Sampling occurred in areas where damage is reported and from outlying areas where this is not the case. Postal-code areas that were rural and strongly affected by damage were oversampled . Residents received letters with personal login codes and one reminder. Eighteen percent (N=4577) signed up for the study, and later received invitations to all questionnaires. Of these 4577, 86% (3934) filled out the first questionnaire."; "Questionnaires were sent via

For peer review only - http://bmjopen.bmj.com/site/about/guidelines.xhtml

				an email link or by post. A reminder was sent after 2 weeks. Participants (T1: N=3934; T5: N=2150) completed measures at 5 time points during 2 years (T1: February 2016, T2: June 2016; T3: November 2016; T4: April 2017; T5: November 2017; see Table 2).
Participants	6	(*a*) *Cohort study*—Give the eligibility criteria, and the sources and methods of selection of participants. Describe methods of follow-up<br>*Case-control study*—Give the eligibility criteria, and the sources and methods of case ascertainment and control selection. Give the rationale for the choice of cases and controls<br>*Cross-sectional study*—Give the eligibility criteria, and the sources and methods of selection of participants	7	
		(*b*) *Cohort study*—For matched studies, give matching criteria and number of exposed and unexposed<br>*Case-control study*—For matched studies, give matching criteria and the number of controls per case		
Variables	7	Clearly define all outcomes, exposures, predictors, potential confounders, and effect modifiers. Give diagnostic criteria, if applicable	8-10	
Data sources/ measurement	8*	For each variable of interest, give sources of data and details of methods of assessment (measurement). Describe comparability of assessment methods if there is more than one group	8-10	
Bias	9	Describe any efforts to address potential sources of bias	10	Analyses controlled for age, gender and education level. Analyses were weighted to correct for sampling effects of age, gender and degree of exposure of postal-code areas . The weights were developed to

For peer review only - http://bmjopen.bmj.com/site/about/guidelines.xhtml

				counteract any potential distortive effect due to age composition, among others (e.g., because younger people were underrepresented, see results section). We report the weighted results. The unweighted results were very similar.
Study size	10	Explain how the study size was arrived at	7	

Continued on next page

For peer review only - http://bmjopen.bmj.com/site/about/guidelines.xhtml

Quantitative variables	11	Explain how quantitative variables were handled in the analyses. If applicable, describe which groupings were chosen and why	10-11	
Statistical methods	12	(*a*) Describe all statistical methods, including those used to control for confounding	10-11	
		(*b*) Describe any methods used to examine subgroups and interactions	10-11	
		(*c*) Explain how missing data were addressed	10	Participants with missing data on the health indices were retained, as multilevel modelling is robust to missingness in estimation of model outcomes. See Table 2 for an overview of the number of participants completing each health measure per time point.
		(*d*) *Cohort study*—If applicable, explain how loss to follow-up was addressed<br>*Case-control study*—If applicable, explain how matching of cases and controls was addressed<br>*Cross-sectional study*—If applicable, describe analytical methods taking account of sampling strategy	11-12	There was attrition during the study. Dropout characteristics revealed no differences between exposed vs. control groups and no association between dropout and health. Analyses showed no indications that attrition influenced any of the effects reported below. Over time, the average age of participants increased, as young people tended to have a higher likelihood of dropout. It is important to note that additional analyses found no significant interaction effect between age and exposure, suggesting that the effects of exposure were age-independent. Because the sample was not entirely representative and attrition relatively high, we carefully checked the potential consequences thereof and found no indications this influenced results.
		(*e*) Describe any sensitivity analyses		
**Results**				

For peer review only - http://bmjopen.bmj.com/site/about/guidelines.xhtml

For peer review only

Participants	13*	(a) Report numbers of individuals at each stage of study—eg numbers potentially eligible, examined for eligibility, confirmed eligible, included in the study, completing follow-up, and analysed		Table 2
		(b) Give reasons for non-participation at each stage	N/A	
		(c) Consider use of a flow diagram	N/A	
Descriptive data	14*	(a) Give characteristics of study participants (eg demographic, clinical, social) and information on exposures and potential confounders		Table 2
		(b) Indicate number of participants with missing data for each variable of interest	N/A	
		(c) *Cohort study*—Summarise follow-up time (eg, average and total amount)		
Outcome data	15*	*Cohort study*—Report numbers of outcome events or summary measures over time		
		Case-control study—Report numbers in each exposure category, or summary measures of exposure		
		Cross-sectional study—Report numbers of outcome events or summary measures		
Main results	16	(*a*) Give unadjusted estimates and, if applicable, confounder-adjusted estimates and their precision (eg, 95% confidence interval). Make clear which confounders were adjusted for and why they were included	13-14	Table 3/4
		(*b*) Report category boundaries when continuous variables were categorized	11	Tables 3/4
		(*c*) If relevant, consider translating estimates of relative risk into absolute risk for a meaningful time period		

Continued on next page

For peer review only

Other analyses	17	Report other analyses done—eg analyses of subgroups and interactions, and sensitivity analyses	
**Discussion**			
Key results	18	Summarise key results with reference to study objectives	15
Limitations	19	Discuss limitations of the study, taking into account sources of potential bias or imprecision. Discuss both direction and magnitude of any potential bias	16-17
Interpretation	20	Give a cautious overall interpretation of results considering objectives, limitations, multiplicity of analyses, results from similar studies, and other relevant evidence	16
Generalisability	21	Discuss the generalisability (external validity) of the study results	17-18
**Other information**			
Funding	22	Give the source of funding and the role of the funders for the present study and, if applicable, for the original study on which the present article is based	19

*Give information separately for cases and controls in case-control studies and, if applicable, for exposed and unexposed groups in cohort and cross-sectional studies.

Note: An Explanation and Elaboration article discusses each checklist item and gives methodological background and published examples of transparent reporting. The STROBE checklist is best used in conjunction with this article (freely available on the Web sites of PLoS Medicine at http://www.plosmedicine.org/, Annals of Internal Medicine at http://www.annals.org/, and Epidemiology at http://www.epidem.com/). Information on the STROBE Initiative is available at www.strobe-statement.org.

For peer review only - http://bmjopen.bmj.com/site/about/guidelines.xhtml
